# Telomere-to-telomere genome assembly of melon (*Cucumis melo* L. var. *inodorus*) provides a high-quality reference for meta-QTL analysis of important traits

**DOI:** 10.1093/hr/uhad189

**Published:** 2023-09-28

**Authors:** Minghua Wei, Ying Huang, Changjuan Mo, Haiyan Wang, Qingguo Zeng, Wenli Yang, Jihao Chen, Xuejun Zhang, Qiusheng Kong

**Affiliations:** National Key Laboratory for Germplasm Innovation & Utilization of Horticultural Crops, College of Horticulture and Forestry Sciences, Huazhong Agricultural University, Wuhan 430070, China; National Key Laboratory for Germplasm Innovation & Utilization of Horticultural Crops, College of Horticulture and Forestry Sciences, Huazhong Agricultural University, Wuhan 430070, China; National Key Laboratory for Germplasm Innovation & Utilization of Horticultural Crops, College of Horticulture and Forestry Sciences, Huazhong Agricultural University, Wuhan 430070, China; National Key Laboratory for Germplasm Innovation & Utilization of Horticultural Crops, College of Horticulture and Forestry Sciences, Huazhong Agricultural University, Wuhan 430070, China; National Key Laboratory for Germplasm Innovation & Utilization of Horticultural Crops, College of Horticulture and Forestry Sciences, Huazhong Agricultural University, Wuhan 430070, China; Hami-melon Research Center, Xinjiang Academy of Agricultural Sciences, Urumqi 830091, China; Hainan Sanya Experimental Center for Crop Breeding, Xinjiang Academy of Agricultural Sciences, Sanya 572014, China; Hami-melon Research Center, Xinjiang Academy of Agricultural Sciences, Urumqi 830091, China; Hainan Sanya Experimental Center for Crop Breeding, Xinjiang Academy of Agricultural Sciences, Sanya 572014, China; National Key Laboratory for Germplasm Innovation & Utilization of Horticultural Crops, College of Horticulture and Forestry Sciences, Huazhong Agricultural University, Wuhan 430070, China

## Abstract

Melon is an important horticultural crop with extensive diversity in many horticultural groups. To explore its genomic diversity, it is necessary to assemble more high-quality complete genomes from different melon accessions. Meanwhile, a large number of QTLs have been mapped in several studies. Integration of the published QTLs onto a complete genome can provide more accurate information for candidate gene cloning. To address these problems, a telomere-to-telomere (T2T) genome of the elite melon landrace Kuizilikjiz (*Cucumis melo* L. var. *inodorus*) was *de novo* assembled and all the published QTLs were projected onto it in this study. The results showed that a high-quality Kuizilikjiz genome with the size of 379.2 Mb and N50 of 31.7 Mb was *de novo* assembled using the combination of short reads, PacBio high-fidelity long reads, Hi-C data, and a high-density genetic map. Each chromosome contained the centromere and telomeres at both ends. A large number of structural variations were observed between Kuizilikjiz and the other published genomes. A total of 1294 QTLs published in 67 studies were collected and projected onto the T2T genome. Several clustered, co-localized, and overlapped QTLs were determined. Furthermore, 20 stable meta-QTLs were identified, which significantly reduced the mapping intervals of the initial QTLs and greatly facilitated identification of the candidate genes. Collectively, the T2T genome assembly together with the numerous projected QTLs will not only broaden the high-quality genome resources but also provide valuable and abundant QTL information for cloning the genes controlling important traits in melon.

## Introduction

Melon (*Cucumis melo* L.) is a globally cultivated horticultural crop with great economic significance. With extensive phenotypic diversity, particularly at fruit level, it was classified into two subspecies based on ovary pubescence, *melo* and *agrestis*, each being further divided into several horticultural groups [1]. Melon is one of the early domesticated fruit crops in the word and has been cultivated for >4000 years [2]. Recently, the comprehensive genome variation map of melon revealed two independent sets of domestication sweeps related to the diverse characteristics of the two subspecies, *melo* and *agrestis* [3]. Compared with subspecies *agrestis*, subspecies *melo* plants generally show thicker flesh of fruits and higher biotic and abiotic stress resistance [4]. Comprehensive evaluation and utilization of the genetic diversity in melon germplasm resources are essential for genetic improvement.

Genomes provide important resources for accelerating genetic improvement. The first reported genome sequence of melon was DHL92 (CM3.5.1) with the size of 375 Mb [5], which was widely used as the reference for multi-omics studies such as epigenome, transcriptome, and genetic mapping since its release in 2012 [4, 6, 7]. A high-quality complete melon genome should cover all the base pairs on the chromosomes in theory. However, owing to the complex structure of the genome and the limitations of short-read sequencing, most of the highly repetitive regions in the first version of the melon genome, involving centromeres, telomeres, and ribosomal DNA (rDNA) sequences, were largely fragmented and incomplete, which greatly limited our understanding of the genome structure and related biological functions [8, 9]. Although the DHL92 genome has been continuously updated, numerous gaps throughout the genome were still unresolved [10, 11]. With the advances in long-read sequencing techniques, scaffolding methods, and assembling algorithms [12–14], several high-quality melon genomes from different botanical groups, such as Payzawat (*C. melo* var. *inodorus*), Charmono (*C. melo* var. *cantalupensis*), Harukei-3 (*C. melo* var. *reticulatus*), and HS (*C. melo* ssp. *agrestis*), were *de novo* assembled and published in recent years [15–18]. These assemblies provided additional references and important information regarding variations in genome organization. However, to provide comprehensive insight into the landscape of genomic variations in melon botanical groups, sequencing and assembling more high-quality telomere-to-telomere (T2T) and gap-free genomes are needed.

The assembled genomes greatly promoted QTL mapping in melon [19, 20]. It is known that most of the economically important traits are controlled by QTLs. Up to now, >60 papers related to QTL mapping in melon have been published. Most of the mapped QTLs are related to fruit traits and disease resistance. However, these QTLs were detected under different environments and genetic backgrounds. The limited population size and large mapping interval made it difficult to determine the candidate genes in the reported studies. Meta-QTL analysis is a powerful approach to identify stable and large-effect QTLs and then to refine their position by integrating the QTL mapping results in multiple studies. Meta-QTL studies have been conducted in several major crops for various traits, such as leaf pigment content in rice [21], root traits in maize [22], grain yield and associated traits in wheat [23], and iron and zinc concentration and content in the seeds of common bean [24]. In melon, the large number of QTL mapping results related to the same trait makes it possible to perform meta-QTL analysis to confirm and narrow down the QTL mapping intervals. Currently, meta-QTL analysis has only been performed on melon fruit shape and size using a limited number of published QTL mapping results [25, 26]. To facilitate mining the candidate genes for QTLs, a more comprehensive meta-QTL analysis is needed for melon by summarizing all the published QTLs.

To address the above issues, in this study a high-quality T2T genome was *de novo* assembled for Kuizilikjiz (*C. melo* L. var. *inodorus*), which is a melon landrace with good flavor and high tolerance to salinity*.* Using the T2T genome as a reference, meta-QTL analysis was performed to integrate the large number of published QTLs in melon. The results not only provide a high-quality genome for genomic research, but also offer valuable resources for cloning the candidate genes responsible for economically important traits in melon.

## Results

### Genome sequencing and assembly

For genome assembly we selected Kuizilikjiz, which is a thick-skinned melon landrace with big fruit, good flavor, and high tolerance to salinity. To gain preliminary information on the genomic characteristics, Illumina sequencing was performed and a total of 26.5 Gb paired-end reads (150 bp) was obtained. The genome size was estimated to be 386.1 Mb based upon the *k*-mer (*k* = 21) frequency distribution (Supplementary Data Fig. S1A). Using the paired-end reads, the chloroplast and mitochondrial genomes and 45S rDNA were successfully assembled (Supplementary Data Table S1).

To assemble the genome, PacBio long-read sequencing was employed, which generated a total of 37.0 Gb HiFi reads with N50 of 19.7 kb (Supplementary Data Table S2). Hifiasm and HiCanu were used to assemble the HiFi reads into contigs. A total of 859 contigs (27 contigs >1.0 Mb) with the total length of 426.5 Mb and N50 of 19.2 Mb were assembled using Hifiasm (Supplementary Data Table S3). Meanwhile, HiCanu assembled the HiFi reads into 1127 contigs (32 contigs >1.0 Mb) with the cumulative length of 448.2 Mb and N50 of 15.4 Mb. Benchmarking universal single-copy orthologs (BUSCO) analysis showed that 98.3 and 98.6% single-copy orthologs were completely detected in the preliminary assembly generated by Hifiasm and HiCanu, respectively (Supplementary Data Table S4).

**Figure 1:**
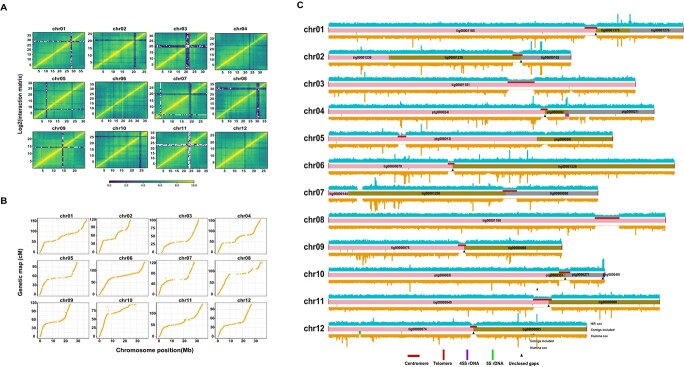
Overview of the Kuizilikjiz genome assembly. **A** Hi-C interaction map for each chromosomal pseudo-molecule at 100 kb resolution. The interaction intensity color bar represents the heat map intensity scale. **B** Collinearity between the genetic map and genome assembly. Scatter plots represent the positions of SNPs in the Kuizilikjiz genome assembly and genetic map constructed using Kuizilikjiz as the female parent. **C** Features of the assembly. The blue shadow at the top represents the coverage density of HiFi reads mapped onto the assembled genome. The rectangles in the middle represent the contigs anchored onto each chromosome. The orange shadow at the bottom represents the coverage density of paired-end reads mapped onto the assembled genome. The red vertical bars at the ends of each chromosome represent the telomeres. The red horizontal bars on the contigs represent centromeres. The purple horizontal bars under the contigs represent the locations of 45S rDNA. The green horizontal bars under the contigs represent the locations of 5S rDNA. The black triangles under the contigs represent the locations of unclosed gaps.

To scaffold the contigs, Hi-C sequencing was performed, generating a total of 52.2 Gb (~135.0×) paired-end reads (150 bp). The valid interaction ratio of Hi-C read pairs determined by HiCUP was 43.0% (Supplementary Data Table S5). The 3D DNA pipeline was used to scaffold the contigs into chromosomal pseudo-molecules based on the Hi-C data. Manual check and correction on contig orientation were performed based on the heat map visualized by JuicerBox. A total of 24 contigs assembled by Hifiasm were anchored onto 12 chromosomal pseudo-molecules with a total length of 374.8 Mb and 15 gaps. Meanwhile, 29 contigs assembled by HiCanu were scaffolded into 12 chromosomal pseudo-molecules with a cumulative length of 381.1 Mb and 17 gaps (Supplementary Data Table S6). Most of the unanchored contigs had lengths smaller than 1.0 Mb. Alignment analysis revealed that 60.0% of the unanchored contigs assembled by Hifiasm and 70.1% of the unanchored contigs assembled by HiCanu were derived from the rDNA sequences and organelle genomes of chloroplasts and mitochondria (Supplementary Data Table S7). To get a more complete genome, the scaffolds assembled by Hifiasm and HiCanu were merged using quickmerge. The merged assembly still had 16 gaps (Fig. 1A).

To validate the merged assembly, a genetic linkage map was constructed using Kuizilikjiz as the female parent of an *F*_2_ genetic population. The *F*_2_ population contained 233 individuals, which were re-sequenced (Supplementary Data Table S8). Using the merged assembly of Kuizilikjiz as a reference, the SNPs were called and genotyped. A high-density genetic map with 12 linkage groups (LGs) and total length of 1497.8 cM were constructed using Lep-Map3. The average genetic distance was 0.38 cM (Supplementary Data Table S9). The orders and locations of the SNPs in the LGs and chromosomes were compared. Highly collinear relationships were observed between the merged assembly and linkage map, demonstrating that the contigs were correctly ordered and oriented at chromosome level (Fig. 1B).

To close the gaps, all the HiFi reads and contigs were aligned to the merged assembly. Then, the sequences matched with two neighboring scaffolds were used to close the gaps. Seven gaps were successfully closed (Supplementary Data Table S10). The chromosomal pseudo-molecules were aligned against the DHL92 v4.0 genome to get the chromosome identifiers. Finally, a chromosome-level genome with the length of 379.2 Mb and nine gaps was successfully assembled for Kuizilikjiz (Fig. 1C, Table 1).

**Table 1:** Statistics of the Kuizilikjiz genome and the other five published melon genomes.

**Assembly name**	**Kuizilikjiz**	**DHL92**	**Harukei-3**	**IVF77**	**Charmono**	**Payzawat**
Genome size^a^ (Mb)	379.2	357.7	370.1	330.7	361.8	363.4
GC (%)	34.7	33.5	33.8	33.5	33.6	33.7
N50 (Mb)	31.7	29.3	30.0	26.8	29.9	30.5
N75 (Mb)	29.4	26.5	27.7	25.1	26.6	27.5
L50	6	6	6	6	6	6
L75	9	9	9	9	9	9
BUSCO (complete single)	1573	1570	1571	1440	1572	1522
BUSCO (complete duplicated)	17	18	17	15	18	37
BUSCO (fragmented)	11	11	11	20	13	14
BUSCO (missing)	13	15	15	139	12	41
BUSCO (genes searched)	1614	1614	1614	1614	1614	1614
BUSCO (complete) (%)	98.6	98.4	98.4	90.1	98.5	96.6
Ns per 100 kb	2.1	32.8	979.4	28.4	986.8	7.1
LAI	11.2	10.4	9.7	9.3	10.9	9.4
QV	54.2	-	-	-	-	-
Number of gaps	9	1174	61	938	423	259

a Genome size represents the total length of chromosome sequences.

### Identification of rDNA, centromeres, and telomeres

Tandem repetitive arrays in genomes mainly include rDNA loci, centromeres, and telomeres, which are the major sources contributing gaps in the genome sequence due to the difficulties in assembling them. To test whether the highly repetitive regions were successfully assembled, the presences of tandem repeats were determined in the genome assembly.

The rDNA sequences were identified by sequence alignment. The assembled 45S rDNA sequence (10 287 bp) was aligned to the Kuizilikjiz genome. Meanwhile, short reads and HiFi long reads were recruited based on the rDNA unit to estimate the copy number of 45S rDNA. The results revealed that there were ~50 copies of rDNA (~514.4 kb) in the genome (Supplementary Data Fig. S1B). The actual copy number of 45S rDNA in the assembled genome was determined by aligning the rDNA sequences with the genome. The results revealed that 93.8% 45S rDNA sequences were successfully assembled in the genome and most of them were located on chr04 (Fig. 1C, Supplementary Data Table S11). Moreover, a 5S rDNA array with the length of 1.8 Mb was found in chr12 (32.25–34.06 Mb, ~578 copies) (Fig. 1C, Supplementary Data Fig. S1C). These results indicated the integrity of rDNA sequences in the genome assembly.

The centromeric regions were defined by *de novo* searching for tandem repeats. A total of 79 345 repeat motifs were identified in the genome. There were ~0.04% repeat motifs with copy number >1000. A large tandem repeat array with the motif length of 354 bp was identified in all chromosomes (Supplementary Data Fig. S1D and E). Meanwhile, the Hi-C interaction heat map also showed that fewer interactions were detected in these regions, demonstrating that these large tandem arrays were centromeres. Sequence analysis showed that lengths of the centromeres ranged from 0.68 to 2.6 Mb in the chromosomes (Fig. 1C, Supplementary Data Table S12).

The telomeric sequences were identified by motif searching in the genome. The TTTAGGG motif array was found in 1698 HiFi reads with the total length of 17.9 Mb (Supplementary Data Table S13). All telomeres were successfully assembled at both ends of the 12 chromosomes. Lengths of the assembled telomeric sequences ranged from 12 to 32 kb with the total of 525 kb and average coverage of ~30× (Supplementary Data Table S14). These results demonstrated that a T2T genome was successfully assembled for Kuizilikjiz.

### Quality assessment of the assembled genome

Contiguity of the assembly was assessed using the number of contigs in each chromosome and long terminal repeat assembly index (LAI). The numbers of contigs assembled in each chromosome varied from 1 to 4 with an N50 value of 31.7 Mb. Particularly, chr03 and chr08 were each represented by a single contig. Only chr10 was scaffolded by four contigs. The remaining chromosomes were represented by two or three contigs. Meanwhile, the LAI distribution was analyzed for each chromosome. There were only three small regions with LAI scores <10, which were located on chr01, chr07 and chr11, respectively (Supplementary Data Fig. S2A). The LAI value at whole genome level was 11.2 (Table 1). The low number of contigs in each chromosome and high LAI value demonstrated the high contiguity of the assembled genome.

Completeness of the assembly was evaluated using the BUSCO value and presences of the tandem repeat arrays. BUSCO analysis was performed for the assembled genome. The result showed that 98.6% of the single-copy orthologs were completely present in the genome assembly, demonstrating the completeness of gene space in the assembly. Moreover, the presences of rDNA sequences, centromeres, and telomeres also indicated the completeness of the repetitive elements in the assembled genome (Table 1).

Accuracy of the assembly was determined using the base-level accuracy and consensus quality value (QV). The Illumina paired-end reads were aligned to the assembly and variants were then called and genotyped. A total of 55 538 homozygous SNPs and 669 homozygous InDels were identified, suggesting the base-level error ratio of 0.02%. Analysis suggested that the QVs for each chromosome varied from 53.5 to 54.9 (Supplementary Data Fig. S2B and C). The extremely low base-level error ratio and high QV demonstrated the high accuracy of the assembly.

Taking these results together, a highly accurate, contiguous, and complete genome was successfully assembled for Kuizilikjiz at T2T level.

### Genome annotation

Transposable elements (TEs) were annotated in the assembled genome. A total of 152.1 Mb TE sequences were identified, accounting for 40.1% of the assembly. LTRs were the most predominant repetitive elements, comprising 27.9% of the whole genome. *Gypsy* and *Copia* were the major retrotransposons, accounting for 15.5 and 10.5% of the total TEs, respectively (Supplementary Data Table S15). DNA transposons accounted for 10.7% of the genome.

**Figure 2:**
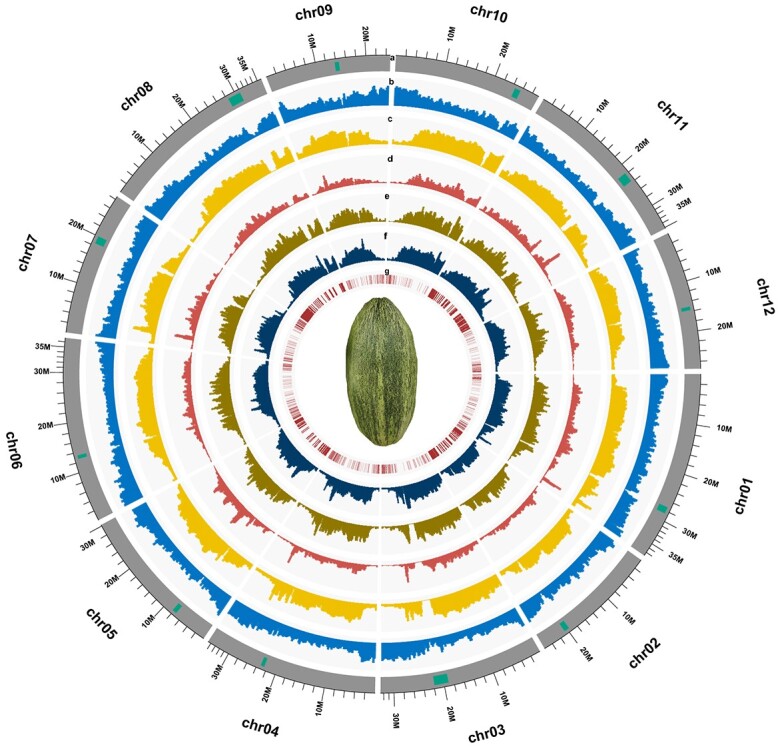
Circos plot of Kuizilikjiz genome annotation. Quantitative tracks (labeled a–g) are aggregated in a 500-kb window. (a) This track represents the chromosome. The highlighted rectangular blocks represent the centromeres. (b) This track represents the density of annotated genes. (c) This track represents the density of repeat sequences. (d) This track represents the density of *Gypsy*. (e) This track represents the density of *Copia*. (f) This track represents the density of transposable elements. (g) This track represents the locations of transcription factors. Photograph in the center shows the fruit of Kuizilikjiz.

Gene prediction and functional annotation were performed for the assembled genome. The gene models were predicted using a combination of *ab initio* search, homology support, and Iso-seq data. A total of 32 693 genes were retained for Kuizilikjiz after prediction and filtering, and 51.9% (16 975 genes) of the predicted genes, corresponding to 33 791 transcripts, were supported by the full-length transcriptome reads. The total gene length was 121.5 Mb with the average gene length of 3.7 kb and coding sequence length of 1.3 kb. The gene models captured 97.1% of the BUSCOs, including 1567 complete genes and 12 missing genes. Functional annotation analysis revealed that 88.8% of the predicted genes had significant matches in at least one database: Nr database (88.4%), Pfam database (62.2%), Swiss-Prot protein database (57.0%), and eggNOG database (75.4%). A total of 5178 (15.8%) genes were annotated as hypothetical proteins and 1532 (4.9%) genes were annotated as uncharacterized proteins. Furthermore, a total of 1314 genes were annotated as transcription factors (TFs) belonging to 58 families. The largest TF family was bHLH (10.1%) followed by MYB (8.3%) (Fig. 2, Supplementary Data Tables S16–S18).

### Comparative genomic analysis

**Figure 3:**
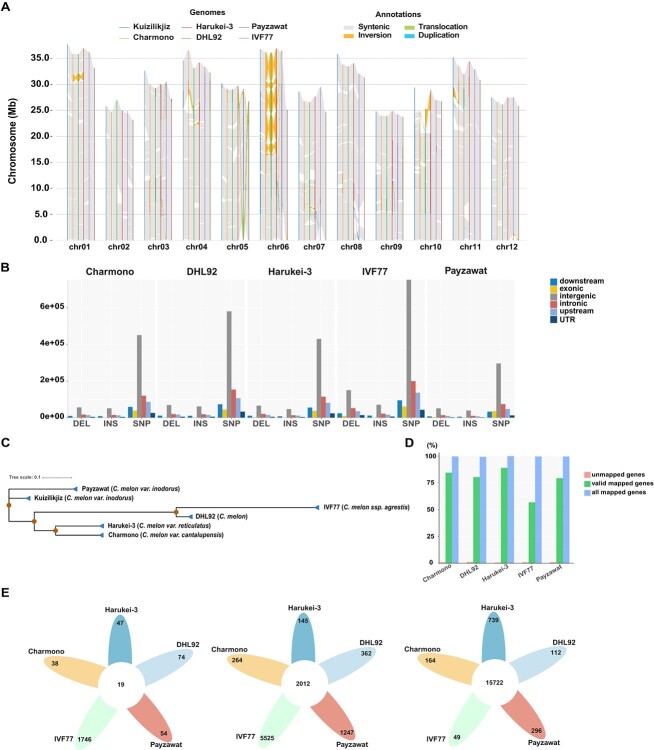
Comparative genomics analysis. **A** Syntenic analysis among the Kuizilikjiz genome and the other five published melon genomes. **B** Numbers and locations of variants detected between Kuizilikjiz and the other five published melon genomes, comprising Charmono, DHL92, Harukei-3, IVF77, and Payzawat. **C** Neighbor-joining phylogenetic tree of Kuizilikjiz genome and the other five published melon genomes with 1000 bootstrap replicates based on SNP data. **D** Percentages of genes lifted from Kuizilikjiz to the other five published melon genomes. All mapped genes include the valid and invalid mapped genes. **E** Flower plots of the unmapped (left), invalid (middle) and valid (right) mapped genes between the Kuizilikjiz genome and the other five published melon genomes.

To assess genome assembly quality, the quality metrics of the Kuizilikjiz genome were compared with those of the other five published melon genomes. The Kuizilikjiz genome had the highest values of N50 and LAI, demonstrating that it had the highest contiguity among the five published melon genomes. Compared with the other melon genomes, the Kuizilikjiz genome also had the highest BUSCO value for complete genes and the smallest number of gaps, indicating its highest completeness. Kuizilikjiz had slightly longer chromosomes than the other genomes except for chr04, which might be attributed to a better assembly of the repeat contents, such as LTRs, telomeres and centromeres (Fig. 3A, Table 1, Supplementary Data Table S19). These results also further suggested better quality of Kuizilikjiz genome assembly.

To explore structural variations (SVs) among melon genomes, Kuizilikjiz was aligned with the five published melon genomes to identify syntenic regions and SVs of translocation, inversion, and duplication. The highest collinearity was observed between Kuizilikjiz and Harukei-3, and the one-to-one syntenic blocks accounted for 93.6% (354.8 Mb) of the Kuizilikjiz genome and 96.9% (358.6 Mb) of the Harukei-3 genome. Meanwhile, a total of 19 inversions and 104 translocations were detected between the two genomes. Alignment between the Kuizilikjiz and Payzawat genomes revealed that 92.8% (351.9 Mb) of the Kuizilikjiz genome matched well with 93.6% (340.3 Mb) of the Payzawat genome, and 10 inversions and 78 translocations were detected between them. Meanwhile, there were 51 and 147 duplication events in the genomes of Kuizilikjiz and Payzawat, respectively. Moreover, several translocations were detected on chr05 between Payzawat and the other five melon genomes. A total of 25 inversions, 35 translocations, and 124 syntenic regions covering >337.0 Mb were identified between the Kuizilikjiz and Charmono genomes. Compared with the DHL92 genome, 35 inversions and 23 translocations as well as 119 syntenic regions covering >320.0 Mb were detected in the Kuizilikjiz genome. The largest inversion occurred on chr06, followed by chr10 in the DHL92 genome. A total of 100 syntenic regions, 33 inversions, and 9 translocations were found between the Kuizilikjiz and IVF77 genomes. Taking these results together, >90% sequences were syntenic among the six melon genomes. Compared with the other five published melon genomes, the large number of SVs in Kuizilikjiz suggested the uniqueness of its genome, which greatly broadens the genome resources of melon (Fig. 3A, Supplementary Data Table S20).

To estimate genome sequence divergence, genetic variants including SNPs, insertions and deletions were called and genotyped between Kuizilikjiz and the other five published genomes. Compared with Kuizilikjiz, a large number of SNPs, ranging from 502 127 (Payzawat) to 1 297 455 (IVF77), insertions, varying from 68 310 (Payzawat) to 131 357 (IVF77), and deletions, in the range of 90 089 (Payzawat) to 288 193 (IVF77), were identified in the other five melon genomes. Most of the variations were located in the intergenic regions followed by the intronic regions. In addition, highly diverged regions, ranging from 3660 (Charmono) to 28 551 (Payzawat), were detected in Kuizilikjiz compared with the other genomes. Variation types of copygains ranging from 20 (Payzawat) to 42 (IVF77), copylosses from 7 (Payzawat) to 39 (IVF77), and tandem repeats ranging from 2 (Charmono and IVF77) to 5 (Payzawat) were also detected between Kuizilikjiz and the other five melon genomes. Phylogenetic tree constructed using the SNPs also indicated that Kuizilikjiz was closely related to Payzawat (Fig. 3B and C, Supplementary Data Table S21).

To reveal the differences in genome annotation, rDNA and gene annotations were compared between Kuizilikjiz and the other melon genomes. The highest copy numbers of 45S rDNA and 5S rDNA were annotated in Kuizilikjiz compared with the other genomes. 18S rRNA, 28S rRNA, and 5.8S rRNA were mainly annotated on chr04 in Kuizilikjiz and Harukei-3. However, they were annotated on chr01 in DHL92 (Supplementary Data Fig. S3A, Supplementary Data Table S22). The length distribution of protein-coding genes in Kuizilikjiz was comparable with that in the other five melon genomes. A higher number of transcripts per gene was observed in Kuizilikjiz, indicating the improvement in annotation of alternative splicing in the present genome (Supplementary Data Fig. S3B and C). Over 98.9% of the annotated genes in the Kuizilikjiz genome were successfully lifted over or mapped to the other five melon genomes, with the valid ORFs ranging from 56.6% (IVF77) to 88.6% (Harukei-3) (Fig. 3D). Among the unmapped genes, 19 genes in Kuizilikjiz could not be mapped to any of the other five genomes. Among the invalid mapped genes, 2012 genes in Kuizilikjiz did not have valid ORFs in any of the other five genomes (Fig. 3E). These consensus unmapped genes and invalid mapped genes were probably the cultivar-specific genes for Kuizilikjiz. Gene Ontology analysis showed that these cultivar-specific genes were significantly enriched in biological processes related to RNA phosphodiester bond hydrolysis, cellular amide metabolic process, floral organ development, response to nutrient, water deprivation, and salt stress (Supplementary Data Fig. S3D). Among the valid mapped genes, 15 722 genes were successfully mapped to all the other melon genomes, suggesting they were the core genes of melon (Fig. 3E).

**Figure 4:**
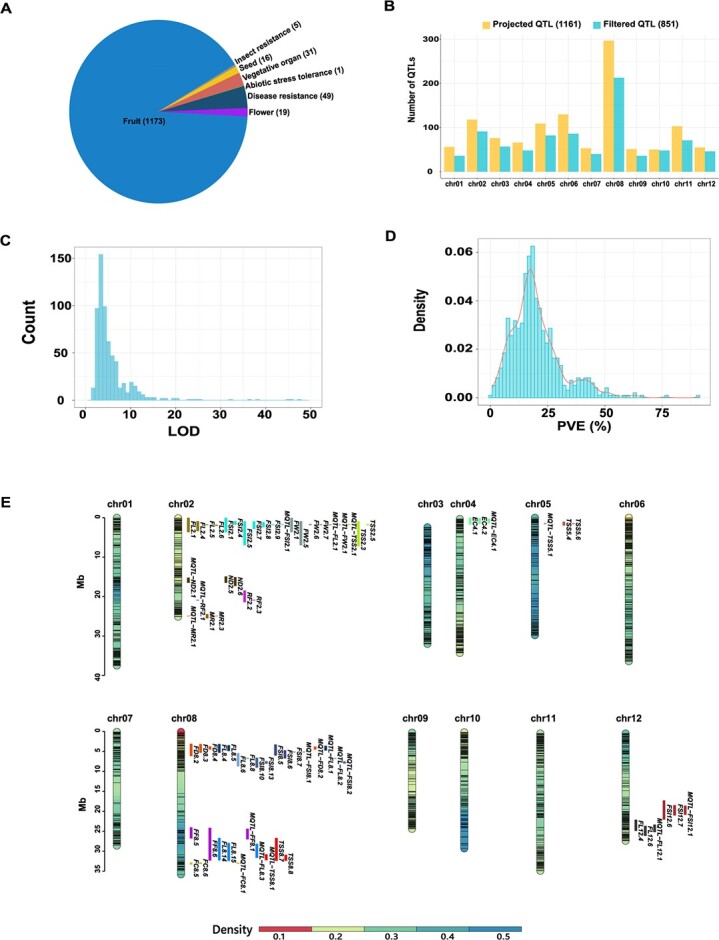
Meta-QTL analysis based on the Kuizilikjiz genome. **A** Numbers and classifications of published QTLs in melon. **B** Numbers and locations of projected and filtered QTLs on the Kuizilikjiz chromosomes. **C** Distribution of LOD scores for all published QTLs in melon. **D** Distribution of PVE (%) for all published QTLs in melon. **E** Locations of the 20 meta-QTLs and their initial QTLs in the Kuizilikjiz genome.

### Projection of published QTLs onto the Kuizilikjiz genome

To integrate all the QTLs mapped in different studies and facilitate candidate gene cloning, the published QTLs in melon were summarized and projected onto the Kuizilikjiz genome. A total of 1294 QTLs were reported in 67 references, which were classified into seven groups, including fruit (1173), flower (19), vegetative organ (31), seed (16), disease resistance (49), insect resistance (5), and abiotic stress tolerance (1) (Supplementary Data Table S23). By sequence alignment, 1161 (89.7%) QTLs were successfully projected onto the Kuizilikjiz genome. QTLs with an interval larger than a quarter of its locating chromosome were excluded, resulting in 851 QTLs for further analysis (Fig. 4A and B, Supplementary Data Table S24). The filtered QTLs were distributed on all 12 chromosomes and were renamed according to their abbreviations and locations. Chr08 had the largest number of QTLs (212) followed by chr02 (91), while chr01 and chr09 had the smallest number of QTLs (36). Average PVE and LOD scores for the QTLs were 19.8% and 6.05, respectively (Fig. 4C and D).

Clusters of QTLs were widely observed in the Kuizilikjiz genome. A cluster region was defined as a 5.0-Mb region harboring more than five QTLs. In total, 47 clusters of QTLs were detected. Among them, 11 clusters of QTLs for volatile organic compounds (VOCs) were identified on seven chromosomes. The chr08 harbored 79 QTLs for VOC, which were grouped into three clusters. These results demonstrated that the genes related to the same metabolic pathway tended to be located closely on the chromosome (Supplementary Data Fig. S4E).

Co-localizations of QTLs for different traits were observed in the Kuizilikjiz genome. Co-localizations of the QTLs for fruit weight (FW) and fruit shape index (FSI) were observed on 9 chromosomes. Five QTLs for chlorophyll degradation (CD), including *CD1.1*, *CD3.1*, *CD6.1*, *CD8.1*, *HD9.1*, and *CD10.1*, were co-localized with the QTLs related to harvest (*PtH1.1*, *PtH3.1*, *HD8.2*, *HD9.1*, and *PtH10.1*). Seven QTLs related to ethylene production or emission, including *DAPE8.1*, *DAPE11.1*, *DAPP8.1*, *ETH3.1*, *ETH3.2*, *ETH8.1*, and *MaxETH8.1*, were also co-localized with the QTLs related to harvest (Supplementary Data Fig. S5). QTLs for soluble solid content (TSS) were always co-localized with QTLs for fructose, glucose or sucrose (SUC). Furthermore, co-localizations were also observed for many QTLs related to biotic and abiotic stresses. For example, *ALB10.1* for resistance to *Alternaria* leaf blight, *DM10.2* for resistance to downy mildew, *WS10.1* for water soaking, and *NEC10.1* for necrosis were co-localized on chr10. In addition, the QTLs for resistance to *Fusarium* rot (*FR8.1*), resistance to *Stemphylium* rot (*SR8.1*), and water soaking (*WS8.1*) were co-localized on chr08. The co-localized QTLs demonstrated that these traits probably had the same genetic architecture or were controlled by pleiotropism (Supplementary Data Fig. S4).

Overlapping of QTLs for the same trait were observed in multiple experiments. A total of 57 overlapped QTLs for 15 traits were detected, including 1 QTL for flesh color (FC), external color (EC), ethylene emission, mottled rind (MR), netting density (ND), rind firmness (RF), and SUC, and 2 QTLs for resistance to powdery mildew (PM), 3 QTLs for carotene (CAR), 3 QTLs for fruit diameter (FD), 4 QTLs for fruit flesh firmness (FF), 6 QTLs for FW, 8 QTLs for TSS, and 9 QTLs for fruit length (FL), as well as 15 QTLs for FSI. Among them, *FSI8.1* was identified in seven independent studies, followed by *FSI2.1*, which was detected by six different studies. *PM12.1* was detected by five studies using different resistance resources, including PMR 6, AR 5, and MR-1. Moreover, six overlapped QTLs, comprising *FW8.1*, *FL2.1*, *FL8.1*, *FSI11.1*, *MR2.1*, and *TSS2.1*, were also identified by four independent studies (Supplementary Data Figs S4 andS5, Supplementary Data Table S25). These overlapped QTLs were stable QTLs, which deserve to receive more attention for fine mapping and cloning of the causal genes.

### Meta-QTL identification and candidate gene mining

Meta-QTL analysis was performed for the QTLs projected onto the Kuizilikjiz genome. Using genetic maps, positions, LOD scores, and PVEs of the QTLs identified under different environmental and genetic backgrounds, a total of 20 meta-QTLs (named MQTLs) were identified, including one MQTL for EC, FC, FD, FF, FW, MR, ND, and RF, and three MQTLs for and TSS, four MQTLs for FSI, and five MQTLs for FL. These MQTLs were distributed on chr02 (7 MQTLs), chr04 (1 MQTL), chr05 (2 MQTLs), chr08 (9 MQTLs), and chr12 (2 MQTLs). The physical intervals of these MQTLs ranged from 0.05 Mb (*MQTL-MR2.1*) to 3.47 Mb (*MQTL-FL8.3*), which were significantly smaller than those of the initial QTLs. The average interval of MQTLs (0.8 Mb) was also 2.5-fold smaller than that of the initial QTLs (2.1 Mb) (Fig. 4E, Supplementary Data Table S26). The number of genes located in the intervals of MQTLs ranged from 6 (*MQTL-MR2.1*) to 295 (*MQTL-FL12.1*) (Supplementary Data Table S27). The average number of genes located in the MQTLs was ~2.8-fold smaller than that of the initial QTLs.

Candidate genes for the MQTLs were predicted using Kuizilikjiz genome annotation. By looking up references, it was found that five MQTLs contained the causal genes whose functions had been validated in melon. These causal genes included *two-component response regulator-like protein APRR2* (*MEL04H000045*) for *MQTL-EC4.1*, *pentatricopeptide repeat-containing (PPR) protein* (*MEL08H001426*) for *MQTL-FC8.1*, and *aminocyclopropane-1-carboxylic acid synthase* (*MEL02M000038*) for *MQTL-FL2.1*, *MQTL-FW2.1*, and *MQTL-FSI2.1*. These results demonstrated the power of meta-QTL analysis in melon. Candidate genes for the remaining MQTLs were further predicted based on function annotations and expression patterns of the genes located in the mapping intervals. Expression data were collected from the Melonet DB database (https://melonet-db.dna.affrc.go.jp/ap/top). Several candidate genes were preliminarily predicted for the other seven MQTLs (Supplementary Data Fig. S6, Supplementary Data Table S28). For example, based on the function annotation and tissue-specific expression pattern, *phenylpropanoid biosynthesis and metabolism* (*MEL08H001292*), *beta-galactosidase* (*MEL08S000376*), *galacturonosyltransferase* (*MEL08H001241*), and *transcription repressor OFP1* (*MEL08H001285*) were predicted as the candidate genes for *MQTL-FF8.1* with high confidence. The predicted candidate genes provided substantial information for cloning and functional validation of the causal genes of MQTLs.

## Discussion

Melon is not only an economically important crop, but also an alternative model plant for elucidating sex determination and fruit ripening [27]. Assembly of a highly continuous and complete reference genome is crucial for genetic and genomic research [28, 29]. However, several published melon genomes were incomplete and none of them achieved the level of T2T, which decreased their values as reference genomes [9, 30]. In addition, a single reference genome is insufficient to capture the full range of genetic diversity in a species [19]. As a result, to improve the quality and broaden the resources of the melon genome, a near-complete T2T genome was *de novo* assembled for melon landrace Kuizilikjiz in this study.

Taking advantage of the complementarity of different assembly strategies and scaffolding approaches facilitated a more contiguous genome assembly. Hifiasm and HiCanu are powerful tools for genome assembly [31, 32]. The contigs assembled by the two tools were merged, resulting in a higher contiguity of assembly compared with that assembled by Hifiasm and HiCanu separately. Hi-C is an efficient approach for scaffolding [33]. However, order and orientation errors of contigs are inevitable when only using Hi-C for scaffolding, which will bring trouble for subsequent analyses, including SV identification. To overcome this problem, a high-density genetic map was constructed and used to correct and validate the Hi-C scaffolding in this study, which significantly increased the correctness of scaffolding. By using the HiFi reads in combination with two assembly tools and two scaffolding methods, a highly contiguous and accurate genome was assembled for the melon landrace Kuizilikjiz.

A T2T genome assembly can uncover structural characteristics of the highly repetitive regions, such as rDNA, centromeres, and telomeres. Ribosomal DNA annotation is a prerequisite for studying the genetic and epigenetic variations of rDNA within and between species [34]. The Mo17 maize genome achieved a complete assembly, which contained ~3000 rDNA copies [35]. However, so far, the rDNA arrays have not been completely assembled in most species, even in the recently published rice and banana genomes [36, 37]. In the Kuizilikjiz assembly, consistent with the results of fluorescence *in situ* hybridization, a large 5S rDNA array (>500 copies) and a nearly intact 45S rDNA array (~50 copies) were assembled on chr12 and chr04, respectively [38]. Although the number of rDNA copies is variable for different melon accessions, the proportions of 18S, 5.8S, and 25S rRNAs resulting from the transcription of complete 45S rDNA should be close to 1:1:1. However, except for Kuizilikjiz and Harukei-3, a significant ratio deviation was observed in the other melon genomes, which was probably due to incomplete assembly of the repeat arrays (Supplementary Data Fig. S3A). Telomeres are regions of high tandem duplication located at both ends of the chromosome. In cucurbitaceous crops, a T2T and gap-free genome was recently assembled for watermelon and a T2T and gap-free assembly was also successful for 6 of 11 chromosomes in bitter melon [39, 40]. However, missing and incomplete telomere sequences were frequently observed in the previously published melon genomes (Supplementary Data Table S19). In this study, all telomeres, with an average length of 21.9 kb, were successfully assembled in the Kuizilikjiz genome. Owing to high repetition and similarity, this is challenging for centromere assembly [8]. The centromeres of all 12 chromosomes were successfully identified with large tandem repeat arrays (354 bp) and most of the centromeres appeared to be complete in Kuizilikjiz, which will greatly facilitate research on the function and evolution of centromeres in melon and other cucurbitaceous crops.

Comparative genomics analysis revealed the high quality and unique features of the Kuizilikjiz genome. Higher genome quality metrics were observed for Kuizilikjiz, indicating that the Kuizilikjiz genome is currently the best assembly with respect to continuity, completeness, and correctness. Charmono (*cantalupensis*) was reported to be closely related to Harukei-3 (*reticulatus*) based on genome divergence degree analysis [15]. A similar result was observed in this study. Except for high synteny, a large number of SVs were also detected between Kuizilikjiz and the other published genomes. Large inversions on chr06 and chr10 were found between Kuizilikjiz and DHL92, which was consistent with the previous comparisons between DHL92 and the other melon genomes [17, 41]. Meanwhile, a large intra-chromosomal translocation occurred on chr05 in Kuizilikjiz when compared with Payzawat. It was reported that a translocation at the tip of chr05 was a difference between thick-skinned melon (Payzawat) and thin-skinned (IVF77 and DHL92) melon [41]. However, like Payzawat, Kuizilikjiz is also a thick-skinned melon landrace. Analysis of telomeric repeat sequences revealed that one of the telomeres was located on chr05 (3 862 320–3 865 229 bp) in Payzawat, which was co-localized with the translocation (Supplementary Data Table S19). As a result, the translocation should be a mis-assembly in Payzawat. Different numbers of genes were annotated in different melon genomes. Except for the effects of different annotation pipelines, genetic diversity is probably the main reason causing the difference in gene annotations. A total of 15 722 core genes and >2000 cultivar-specific genes were identified among the six melon genomes in this study. The large number of cultivar-specific genes and SVs not only demonstrated the uniqueness of the Kuizilikjiz genome, but also proved the great value of the Kuizilikjiz genome in broadening the genetic diversity of the current melon genome resources.

Projection of the published QTLs onto the high-quality Kuizilikjiz genome was an efficient method to integrate all the QTLs identified in melon. In this study, 1294 QTLs from 67 studies were collected and 1161 (89.7%) QTLs were successfully projected onto the Kuizilikjiz genome. Integration of QTLs on the Kuizilikjiz genome provided a comprehensive summary of the QTLs identified in melon. Meta-QTL analysis is a powerful strategy for refining the confidence intervals by integrating QTLs independently identified in different studies. The first meta-QTL study in melon was reported in 2014 mainly involving meta-QTLs for fruit shape and fruit weight [26]. Recently, 158 consensus QTLs associated with fruit size, fruit shape, and fruit weight were identified in the Cucurbitaceae family [25]. However, many of the consensus QTLs were identified in a single study or genetic background and the mapping intervals for most of them were still very large. In this study, a genome-wide meta-analysis approach was employed to summarize all the published QTLs in melon, resulting in 20 meta-QTLs. In bread wheat, a comprehensive meta-QTL analysis was conducted on 2230 QTLs of yield-related traits and 76 core meta-QTLs with a physical distance <25 Mb were detected [42]. Similarly, 11 and 34 meta-QTLs related to grain drying rate and grain water content, respectively, were identified from 282 QTLs in maize [43]. All the above studies demonstrated that the mapping interval and number of candidate genes were significantly reduced in meta-QTLs compared with their initial QTLs.

The identified meta-QTLs greatly facilitated prediction of the candidate genes. Several genes with functions validated in melon were found in the mapping intervals of meta-QTLs. For example, *CmPPR1* [44] affecting carotenoid accumulation and *CmAPRR2* [45] responsible for rind and flesh pigment intensity were annotated in the mapping intervals of *MQTL-FC8.1* and *MQTL-EC4.1*, respectively. These results demonstrated the effectiveness of meta-QTL analysis. Several candidate genes for the meta-QTLs could be predicted according to the gene annotation of the Kuizilikjiz genome. Flesh firmness is an important fruit quality trait affecting consumer preference. A meta-QTL related to flesh firmness (*MQTL-FF8.1*) was identified on chr08. In the mapping interval of *MQTL-FF8.1*, *MEL08H001285*, encoding a transcription repressor, might be the candidate gene, because its orthologs of *MaOFP1* and *AtOFP1* were reported to control texture firmness and regulate secondary cell wall formation in *Arabidopsis* [46, 47]. The netting feature is an important factor affecting the external appearance of melon fruits. Biosynthesis of skin compounds such as cutin and epicuticular waxes as well as flavonoids was reported to be dramatically affected at the onset of reticulation in melon [48]. A meta-QTL for netting density (*MQTL-ND2.1*) was identified on chr02 in this study. *MEL02H000654* and *MEL02M000346* were detected in the mapping interval of *MQTL-ND2.1*. The orthologous genes of *MEL02M000346* in *Oryza sativa* (*WSL4*) [49], *Brassica rapa* (*BrWAX3*) [50], *Medicago truncatula* (*WFL*) [51], and *Arabidopsis* (*KCS11*) [52] were demonstrated to participate in cuticular wax biosynthesis. Meanwhile*, SAGL1*, the orthologous gene of *MEL02H000654*, was reported to play an important role in the regulation of phenylpropanoid biosynthesis in *Arabidopsis* [53]. *SAGL1* acted as a post-translational regulator for phenylalanine ammonia-lyase and mutations in *SAGL1* caused high accumulation of lignin [54]. Transcriptome analysis showed that the putative cutin and wax biosynthetic genes were suppressed, while genes for phenylpropanoid biosynthetic and suberin deposition were highly active in reticulated melon skin [48]. As a result, *MEL02H000654* and *MEL02M000346* were probably the candidate genes for *MQTL-ND2.1*. These results demonstrated that the reduced mapping interval obtained with meta-QTL analysis in combination with the high-quality genome assembly and annotation of Kuizilikjiz significantly increased the efficiency of candidate gene prediction in melon.

Taking these results together, a T2T genome was *de novo* assembled for melon in this study, which not only provides a high-quality reference genome but also broadens the genetic diversity of melon genome resources. Moreover, the QTLs projected onto the Kuizilikjiz genome and the identified meta-QTLs will greatly facilitate prediction and cloning of the candidate genes controlling important traits in melon.

## Materials and methods

### Plant materials

Kuizilikjiz (*C. melo* L. var. *inodorus*), a thick-skinned melon landrace with good flavor, large fruit size, and high tolerance to salinity, was selected for genome assembly. The inbred line of Kuizilikjiz was obtained by self-pollination for over seven generations and was used for sequencing. Using Kuizilikjiz as the female parent and inbred line NY-1 as the male parent, an *F*_2_ population consisting of 233 plants was constructed and used for genetic map construction.

### Genomic DNA extraction and sequencing

The young and fresh leaves were collected from Kuizilikjiz seedlings and immediately frozen in liquid nitrogen. Using a Plant Genomic DNA Kit (Tiangen, Beijing, China), high-quality genomic DNA was extracted. For the genome survey, using the NEBNext^®^ Ultra DNA Library Prep Kit for Illumina, the library was constructed and then sequenced on an Illumina HiSeq 2000 instrument. For HiFi sequencing, using a SMRTbell Express Template Prep Kit 2.0, a single-molecule real-time (SMRT) sequencing library was developed and then sequenced on a PacBio Sequel II SMART cell using the circular consensus sequence method. For Hi-C sequencing, the sample was fixed with formaldehyde and then used to develop a sequencing library. DpnII was used to digest the cross-linked nuclei. The Hi-C library was sequenced using an Illumina HiSeq 2000 instrument and then 150-bp paired-end reads were generated. For whole-genome re-sequencing, the young leaves were separately sampled from the parental lines and *F*_2_ individuals. Genomic DNA was also isolated using the method described above. Sequencing libraries were constructed and then sequenced on a DNBSEQ-T7 platform (BGI).

### RNA extraction and isoform sequencing

The fresh tissues, including roots, stems, leaves, flowers, and fruits at 20 and 35 days after pollination, were sampled and immediately frozen in liquid nitrogen. Then, using TRIzol reagent (Invitrogen), total RNAs were isolated from each sample. Equal amounts of the RNA samples were mixed. Using a SMARTer^®^ PCR cDNA Synthesis Kit (Takara Biotechnology, Dalian, China), reverse transcription was performed on the mixed RNA. Using a Pacific Biosciences DNA Template Prep Kit 2.0, the SMRTbell library was developed and then sequenced on a PacBio Sequel II platform.

### Genome assembly pipeline

For the genome survey, 21-bp *k*-mer frequency was counted based on the paired-end reads using Jellyfish (2.2.10), and then Genomescope 2.0 was employed to estimate the genome size and heterozygosity [55, 56]. For genome assembly, the HiFi reads were assembled using Hifiasm (0.16.1-r375) with default parameters and HiCanu (v2.2) with parameters of genomeSize = 380 m, minInputCoverage = 0, useGrid = false, and -pacbio-hifi, respectively [31, 32]. GetOrganelle v1.7 was used to assemble the organelle genome [57].

Hi-C data were used for scaffolding. The valid interaction pairs were identified by HiCUP (v0.9.2) with default parameters [58]. The Juicer and 3D-DNA *de novo* genome assembly pipeline was employed to scaffold the contigs [59, 60]. The mis-ordered and mis-oriented contigs were adjusted manually and validated using Juicebox (1.11.08) [60]. All unanchored contigs were subsequently aligned to the mitochondrial and chloroplast genomes by minimap2 (2.22-r1101) to determine their origins [61]. Finally, the assemblies were merged by quickmerge v0.3 using HiCanu assembly as reference and Hifiasm assembly as query [62]. Telomeres and centromeres were found based on previously developed scripts (https://github.com/VGP/vgpassembly/) and Tandem repeats finder [63].

Completeness of the Kuizilikjiz genome was assessed using QUAST v5.0.2 and BUSCO (v5.4.0) with the library of embryophyta_odb10 [64, 65]. To validate the continuity of the assembled genome, LAI was calculated using LTR_retriever with the default settings [66]. Merqury (v1.3) based on the analysis of 21-mer spectra was employed to evaluate completeness of the assembly using PCR-free Illumina and HiFi reads [67].

### SNP calling and genetic map construction

Using fastp, quality control was performed on the whole-genome re-sequencing data, which were than mapped onto the Kuizilikjiz genome using BWA-MEM (v0.7.17) [68, 69]. The submodules of BCFtools, including mpileup and call, were selected to identify the variants [70]. Using lep-MAP3, the genetic linkage map was set up based on SNPs [71]. Briefly, the ParentCall2 submodule was used to call the parental genotypes, and then the Filtering2 submodule was used to remove the loci with missing genotype ratio >0.2. The remaining SNPs were separated into multiple LGs based on the LOD scores (ranging from 25 to 45). Finally, the markers were ordered in each LG, and the OrderMarkers2 submodule was employed to calculate Kosambi genetic distances between the adjacent markers. The SNPs without recombination at the interval were clustered into a bin.

### Genome annotation pipeline

Repetitive sequences and families were identified using EDTA v2.0.1 and RepeatModeler v2.0.4 [72]. Then, repetitive regions of the genome were soft-masked using RepeatMasker v4.1.4 [73]. Gene structures were predicted with evidence from *de novo* prediction, homology protein sequences, and Iso-seq sequences. The dataset of *de novo* prediction was obtained using Augustus v3.2.3 [74]. For homology-based prediction, the protein data were collected from the published melon genomes (http://cucurbitgenomics.org/v2/). Protein sequences were clustered using cd-hit (v4.8.1) (−c 0.99 -d 0 -M 0 -g 1) to filter redundant sequences [75]. The BEAKER2 annotation strategy was implemented for protein homolog annotation [76]. For Iso-seq-based prediction, a total of 39 036 780 raw subreads (~70.3 Gb) with an average length of 1800 bp were obtained. Using a circular consensus sequencing (CCS) algorithm for intramolecular error correction, a total of 827 725 highly accurate CCS reads were generated. The polished CCS reads were further passed to the lima tool (https://lima.how/) to demultiplex and remove barcodes and primer sequences. The refine tool was then used to trim poly(A) tails and filter concatemers from the full-length reads, resulting in a total of 615 426 full-length non-chimeric (FLNC) reads (Supplementary Data Table S29). Finally, the FLNC reads were collapsed into unique isoforms. The PASA pipeline was applied to predict genes based on the isoforms [77]. EVidenceModeler was employed to compute weighted consensus gene structure annotations based on the prediction results obtained by the above three methods to build a set of high-confidence genes [78]. These data sets were further optimized and integrated.

Given the potential false positives of the gene prediction, several filtering steps were taken. Single-exon genes with only *ab initio* prediction evidence and without homology or expressions were discarded. Protein domains on the annotated genes were identified by searching the databases of Swiss-Prot, Nr, and Pfam using BLASTP (E-value <1e−5) and HMMER. In cases where there were no homologies in any of these databases, the genes were excluded. Frameshifted and partial genes were filtered using GffRead v0.12.1 [79]. Finally, the final gene models were functionally annotated by searching the Swiss-Prot, eggNOG-mapper, Pfam, and Nr databases. The Barrnap tool (v0.9) was employed to predict rRNA genes (https://github.com/tseemann/barrnap). Transcription factors were predicted by plantTFDB [80].

### Comparative genomic analysis

Genomes of Payzawat [17], DHL92 [11], IVF77 [41], Harukei-3 [16], and Charmono [15] were obtained from the Cucurbit Genomics Database (http://cucurbitgenomics.org/v2/), and then aligned to the Kuizilikjiz genome by minimap2. SyRI was employed to detect SVs with the default parameters [81]. The variants found by SyRI were divided into two hierarchies, including genome structure rearrangements and genome sequential variations. Liftoff was used to lift over genes from Kuizilikjiz to the other melon genomes [82]. The phylogenetic tree was constructed using Phylip and IQ-TREE [83, 84].

### QTL collection and meta-QTL analysis

The published QTLs in melon were manually collected by retrieving the PubMed and Web of Science databases using QTL and melon as the keywords. To avoid data redundancy, only the QTLs identified by experiments were collected. The metadata about QTLs were summarized, including QTL name, LOD, PVE, method of analysis, parent lines, and population type and size. The sequences flanking the mapping interval of each QTL were extracted with the length of 1 kb according to the corresponding reference genome. Then, the flanking sequences were aligned to the Kuizilikjiz genome using minimap2 to locate the QTLs. In this way, the published QTLs were projected onto the Kuizilikjiz genome. The projected QTLs were named according to their chromosome locations and trait names. The vocabularies reported in cucumber were used as a reference to describe the quantitative traits of melon [85]. Finally, the genetic map and QTL information were used as input files to perform the meta-QTL analysis by the Biomercator v4.2 via the Veyrieras two-step algorithm [86]. The optimal MQTL model was determined using the Akaike information criterion (AIC), and the lowest AIC value indicated the best fit.

## Acknowledgements

This work was supported by the Key R&D Project of Hubei Province (2021BBA101), the Fundamental Research Funds for the Central Universities (2662020YLPY024), the Key R&D Project of Xinjiang Academy of Agricultural Sciences (xjkcpy-2022006), the Tianshan Innovation Team (2022D14015), and the China Agriculture Research System (CARS-25).

## Author contributions

Q.K. and X.Z. conceived the project. W.Y. and J.C. prepared the materials. M.W., Y.H., C.M., H.W., and Q.Z. performed the data analysis. M.W. drafted the manuscript. Q.K. revised the manuscript. All authors have read and approved the final version of the paper.

## Data availability

All raw sequencing data generated in this project, including HiFi, Hi-C, and Illumina, were deposited at NCBI (https://www.ncbi.nlm.nih.gov/) under BioProject accession numbers PRJNA979338 and PRJNA952861. The assembly and annotation files were submitted to the National Genomics Data Center (https://ngdc.cncb.ac.cn/). The accession number for the Kuizilikjiz genome assembly reported in this paper is SAMC2920780 under the project PRJCA018418.

## Conflict of interest

The authors declare that they have no conflicts of interest.

## Supplementary data

Supplementary data is available at *Horticulture Research* online.

## Supplementary Material

Web_Material_uhad189
