# Supplementary figures and images for: Telomere-to-telomere genome assembly of melon (Cucumis melo L. var. inodorus) provides a high-quality reference for meta-QTL analysis of important traits
Source: Hortic Res. 2023 Sep 28;10(10):uhad189. doi: 10.1093/hr/uhad189 (PMC10615816; doi:10.1093/hr/uhad189)

A

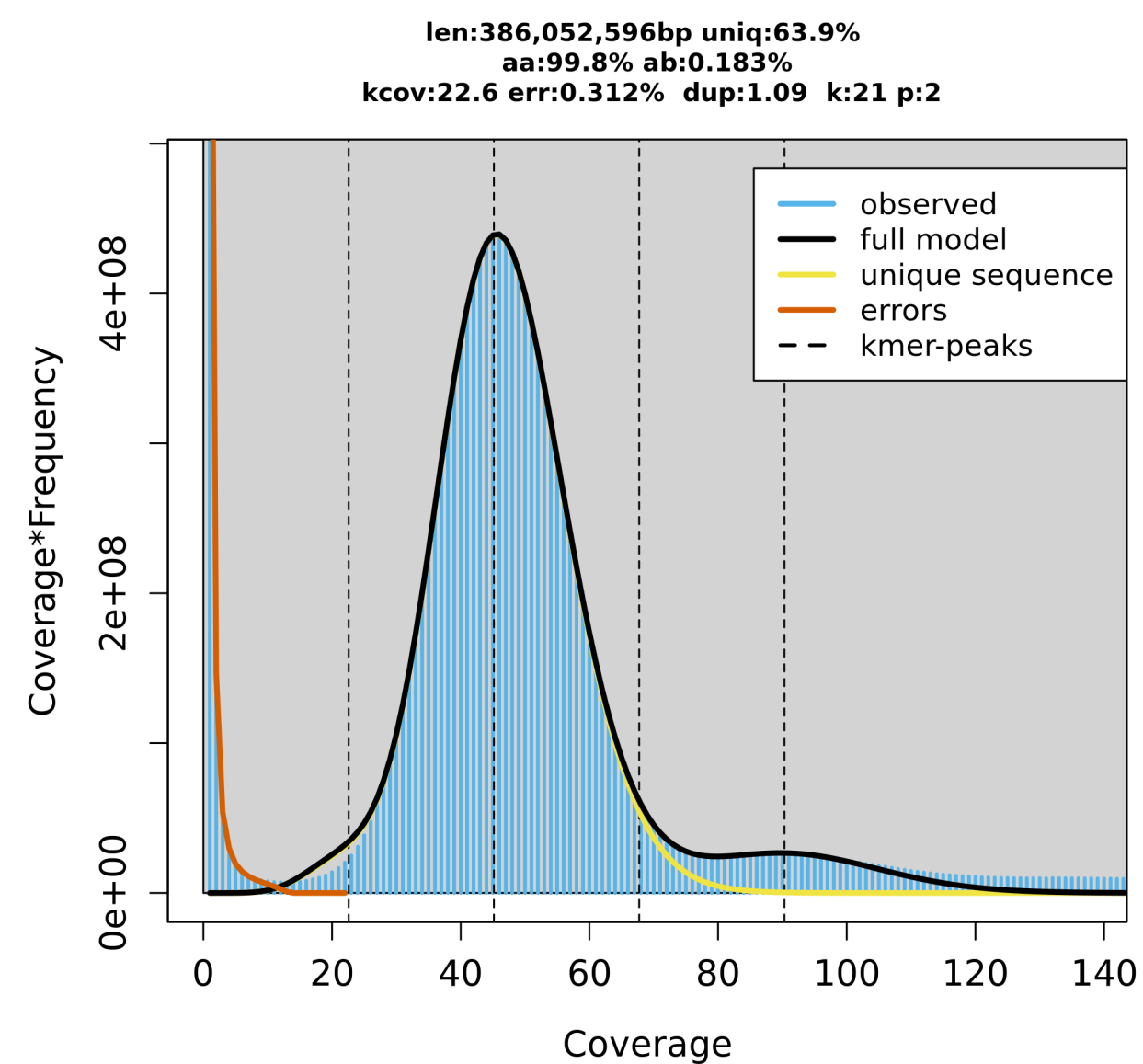

B

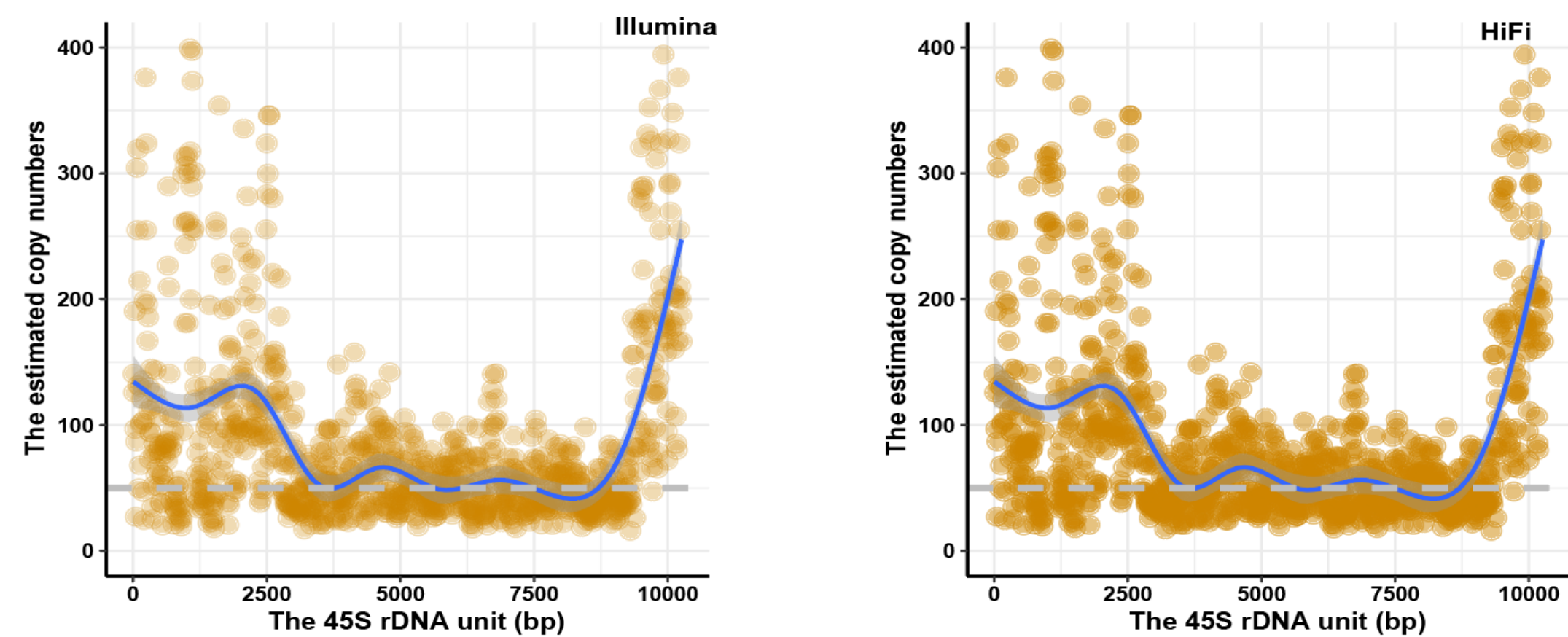

C

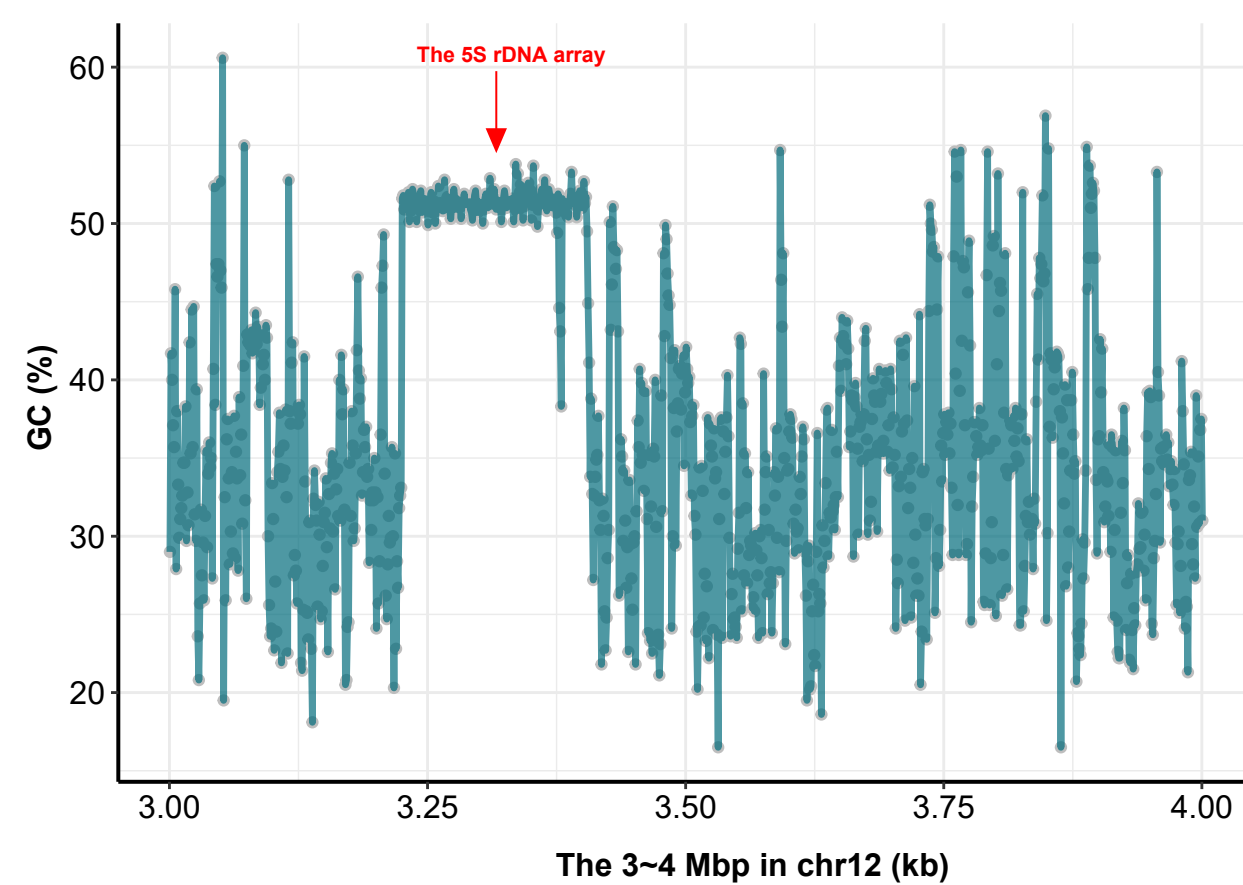

D

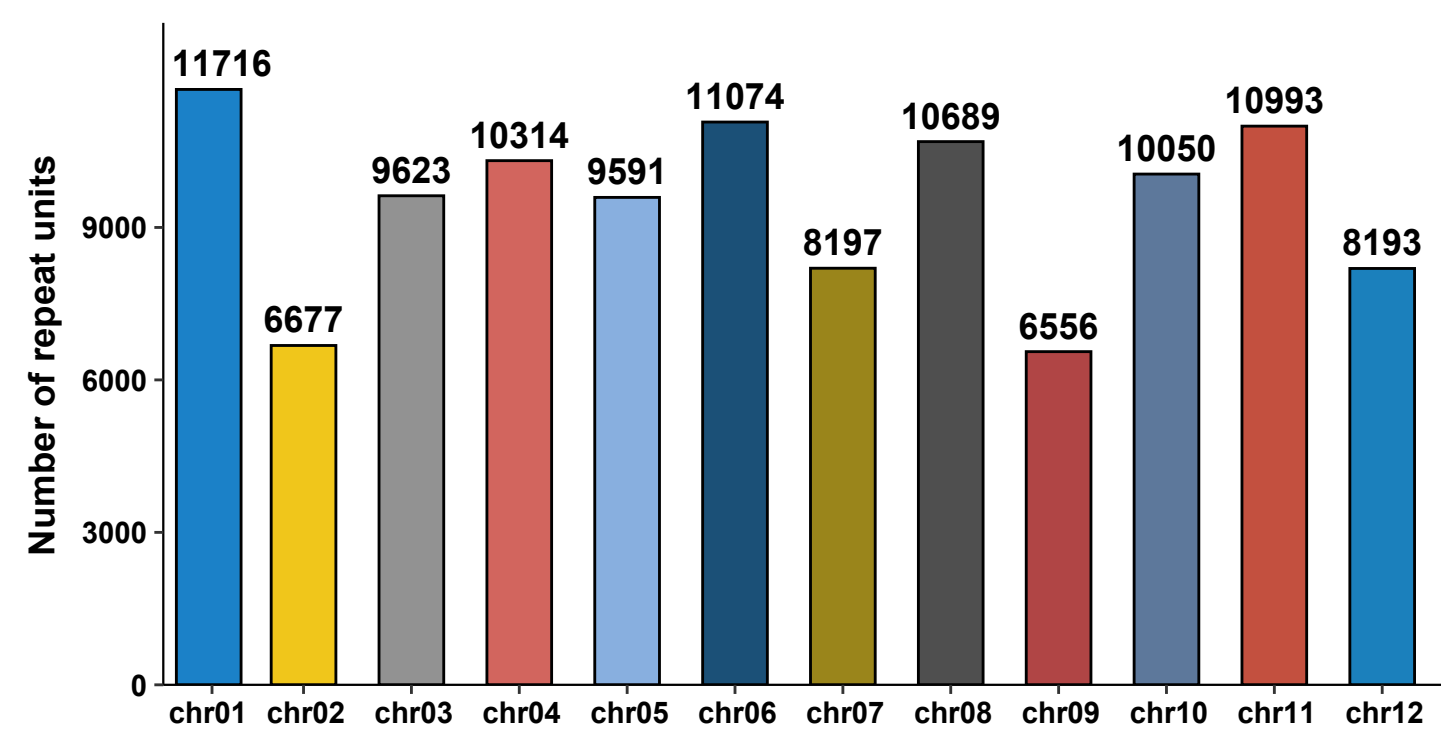

E

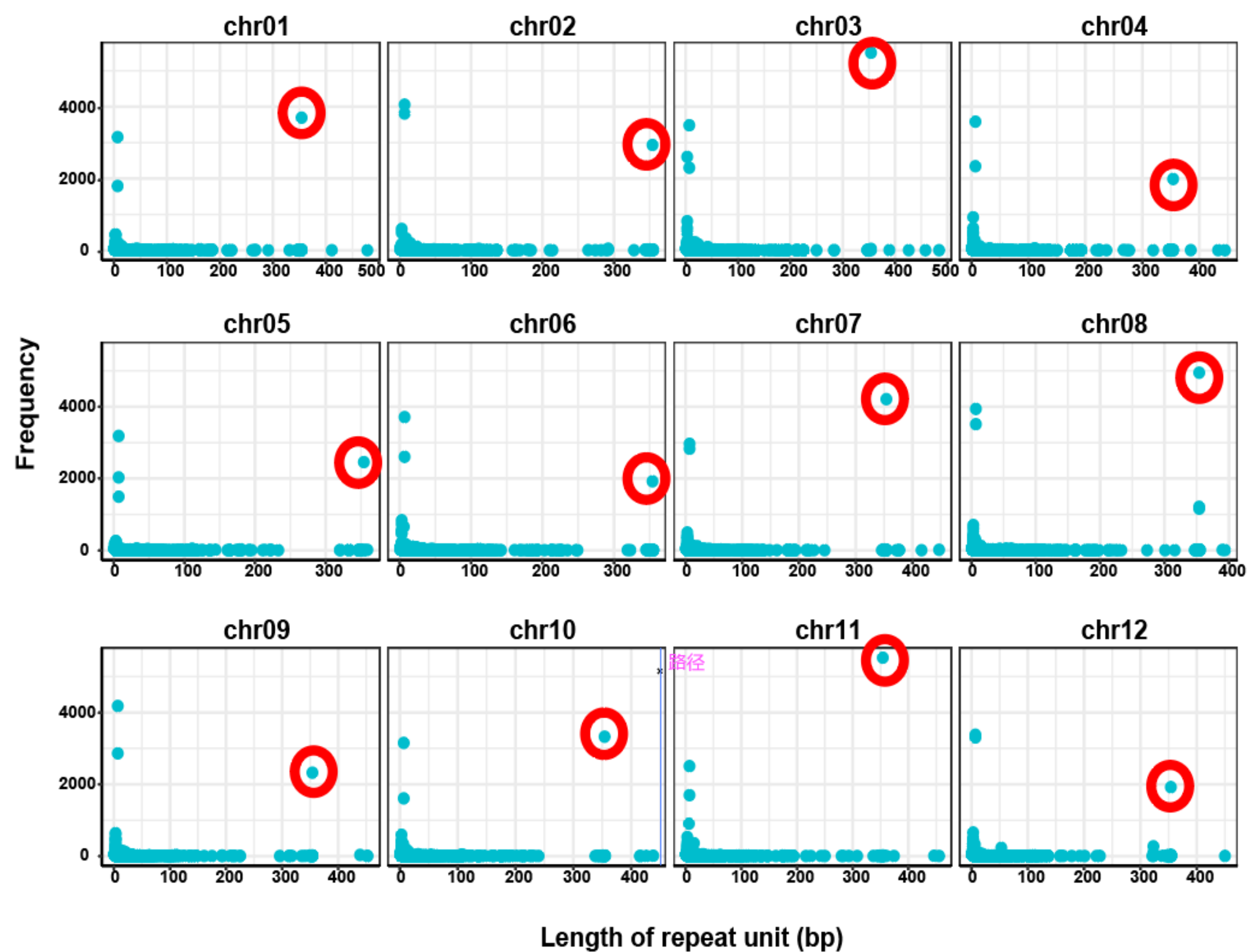

Supplement: Web_Material_uhad189 [file web_material_uhad189.zip › Figure S1.pdf]

A

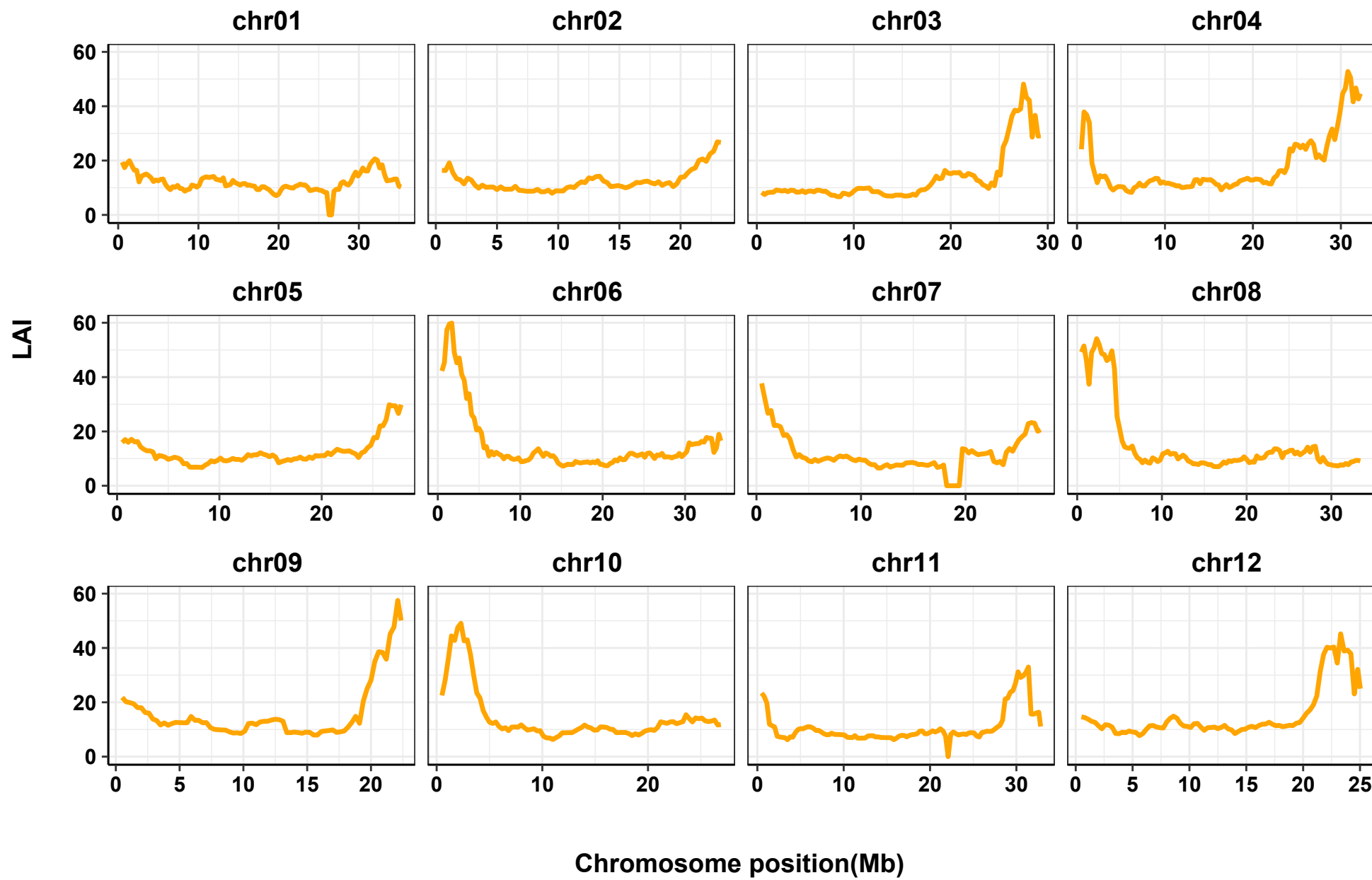

B

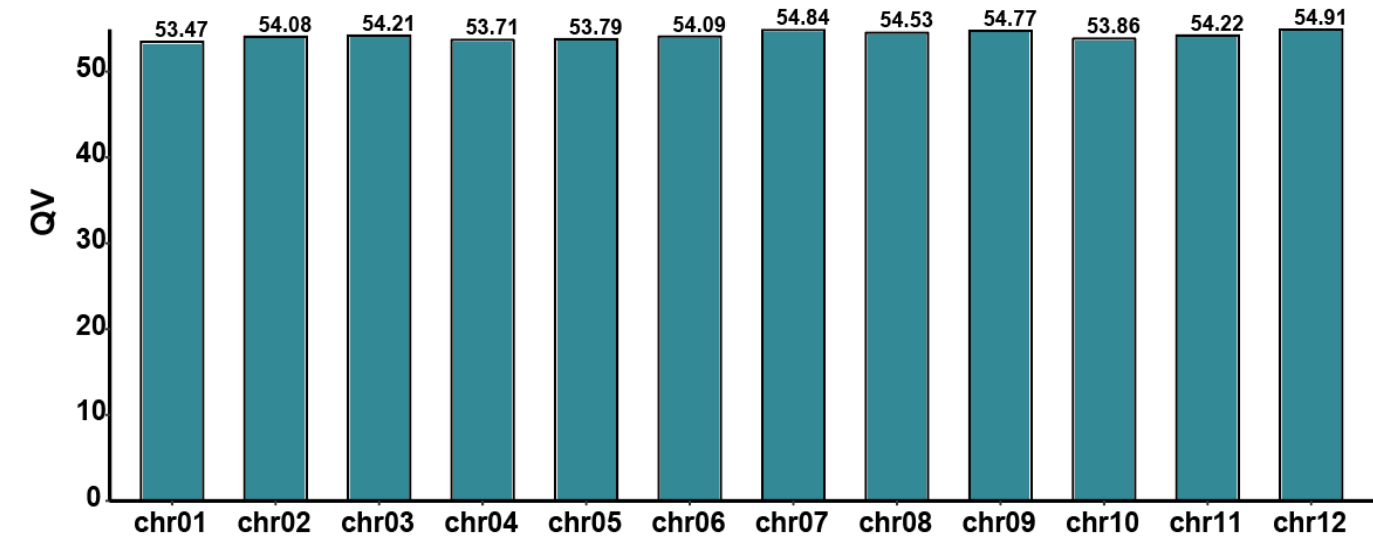

C

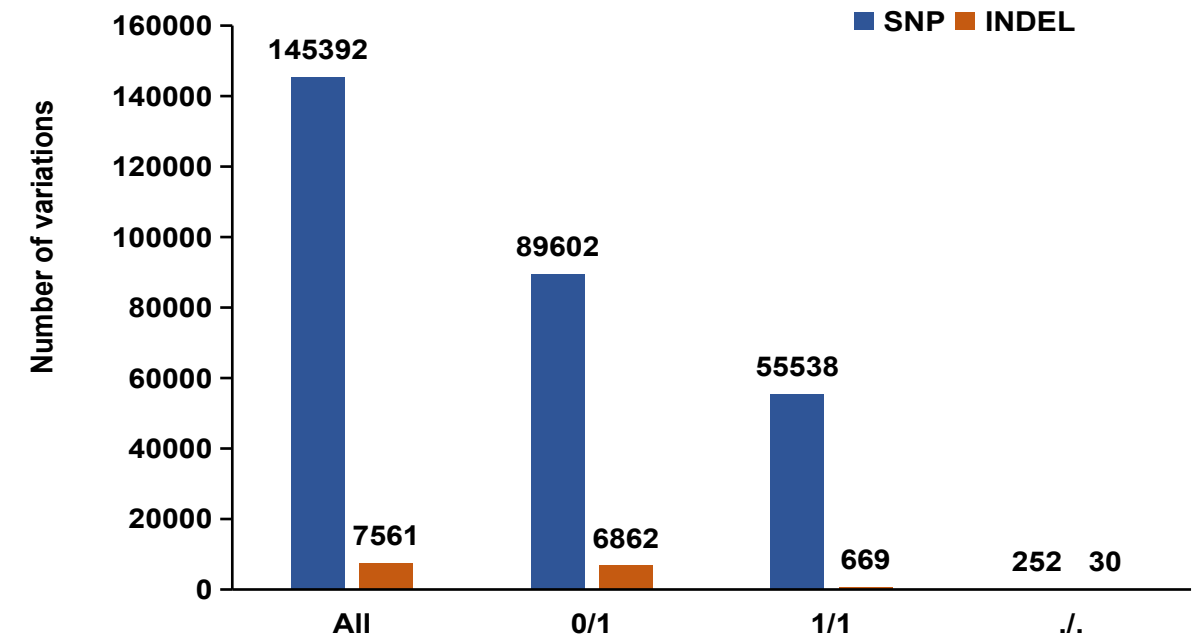

Supplement: Web_Material_uhad189 [file web_material_uhad189.zip › Figure S2.pdf]

A

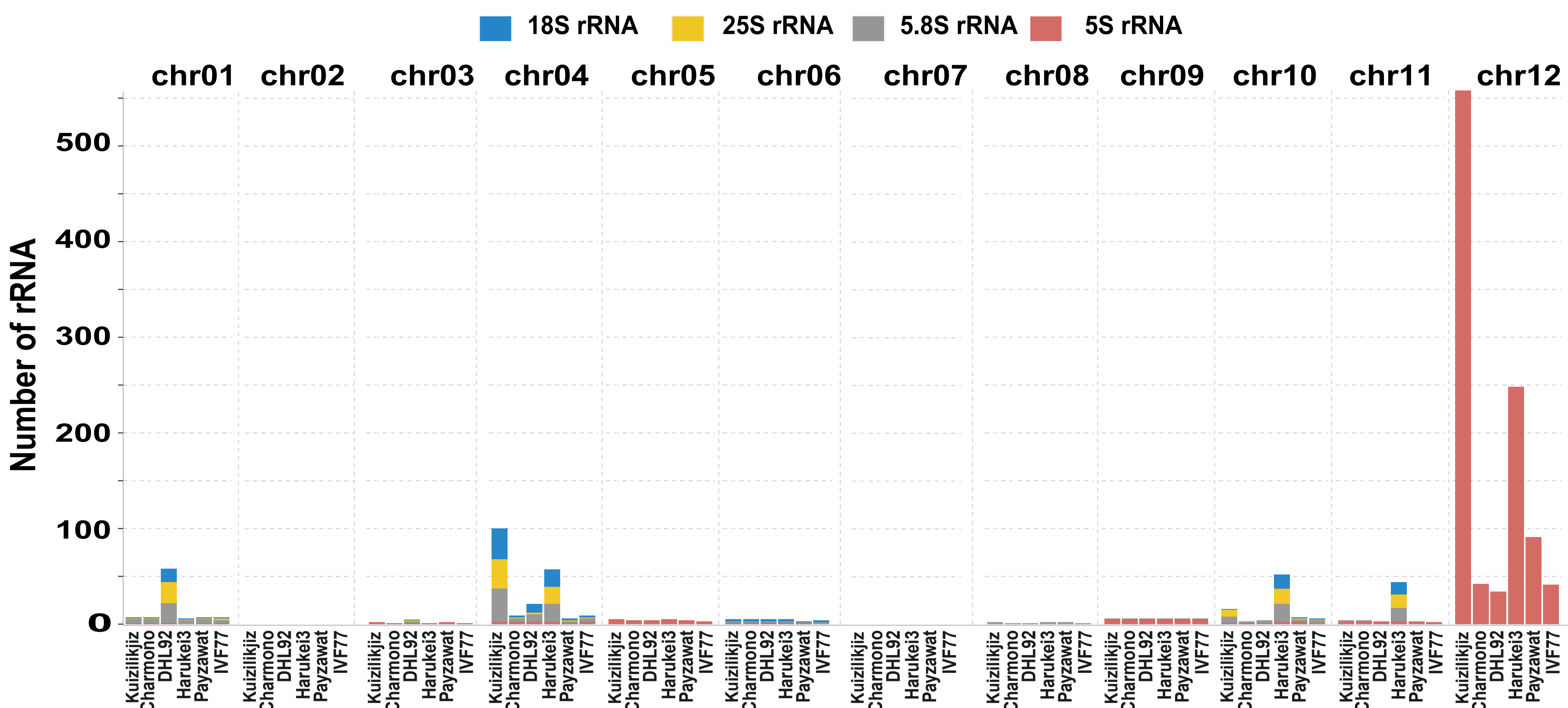

B

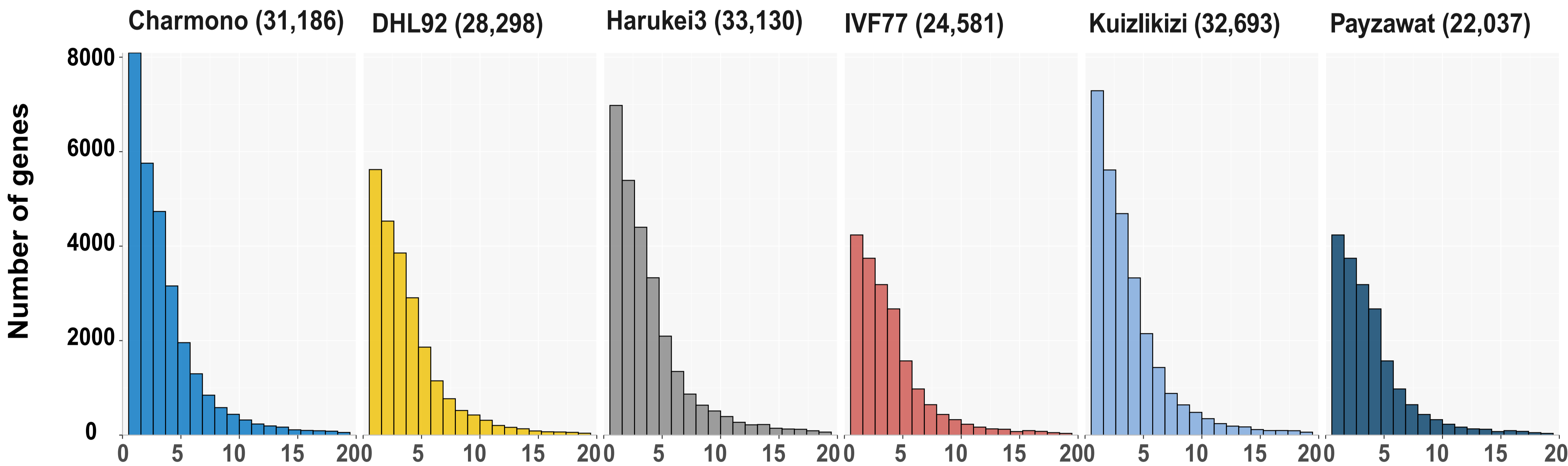

C

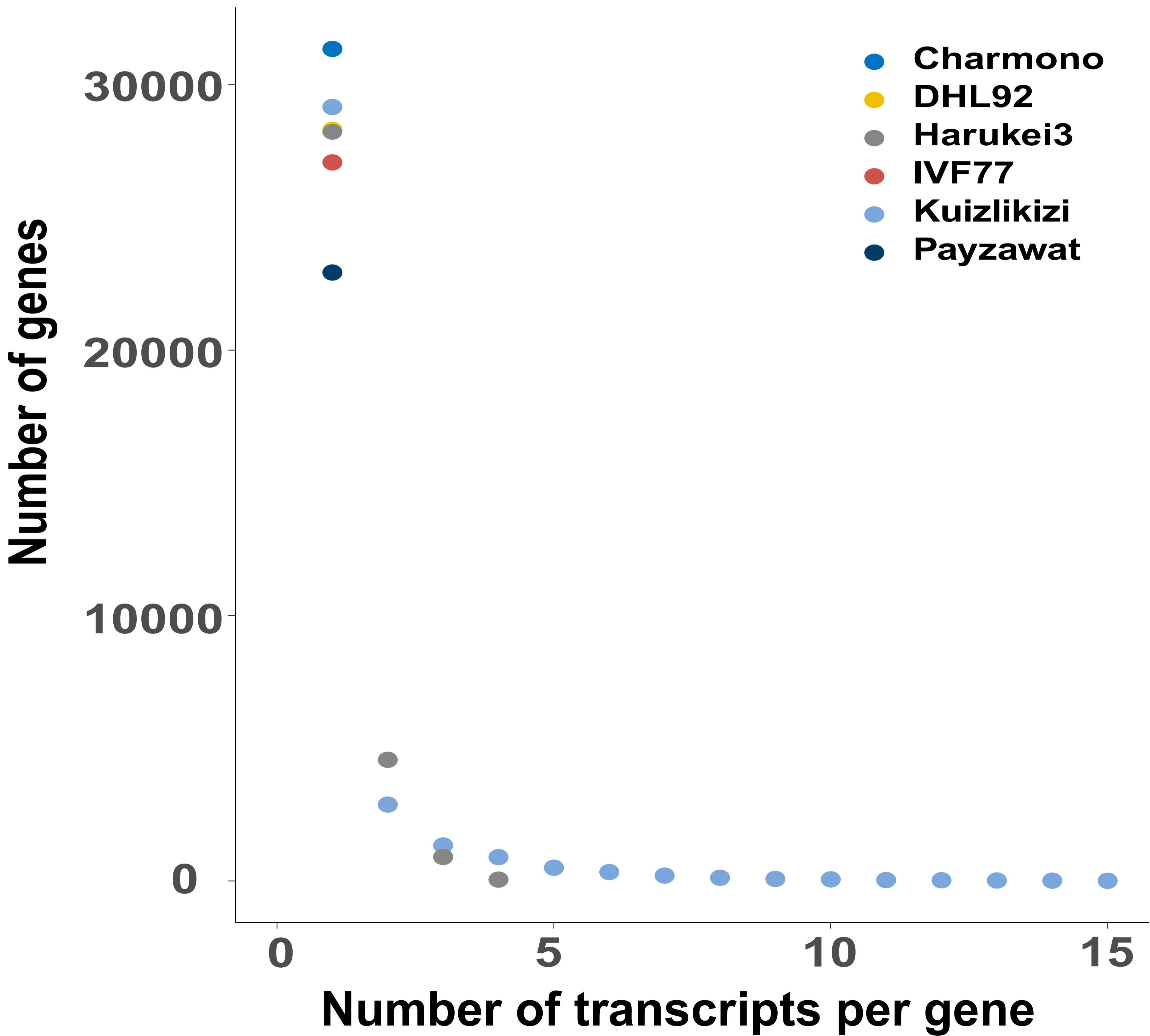

D

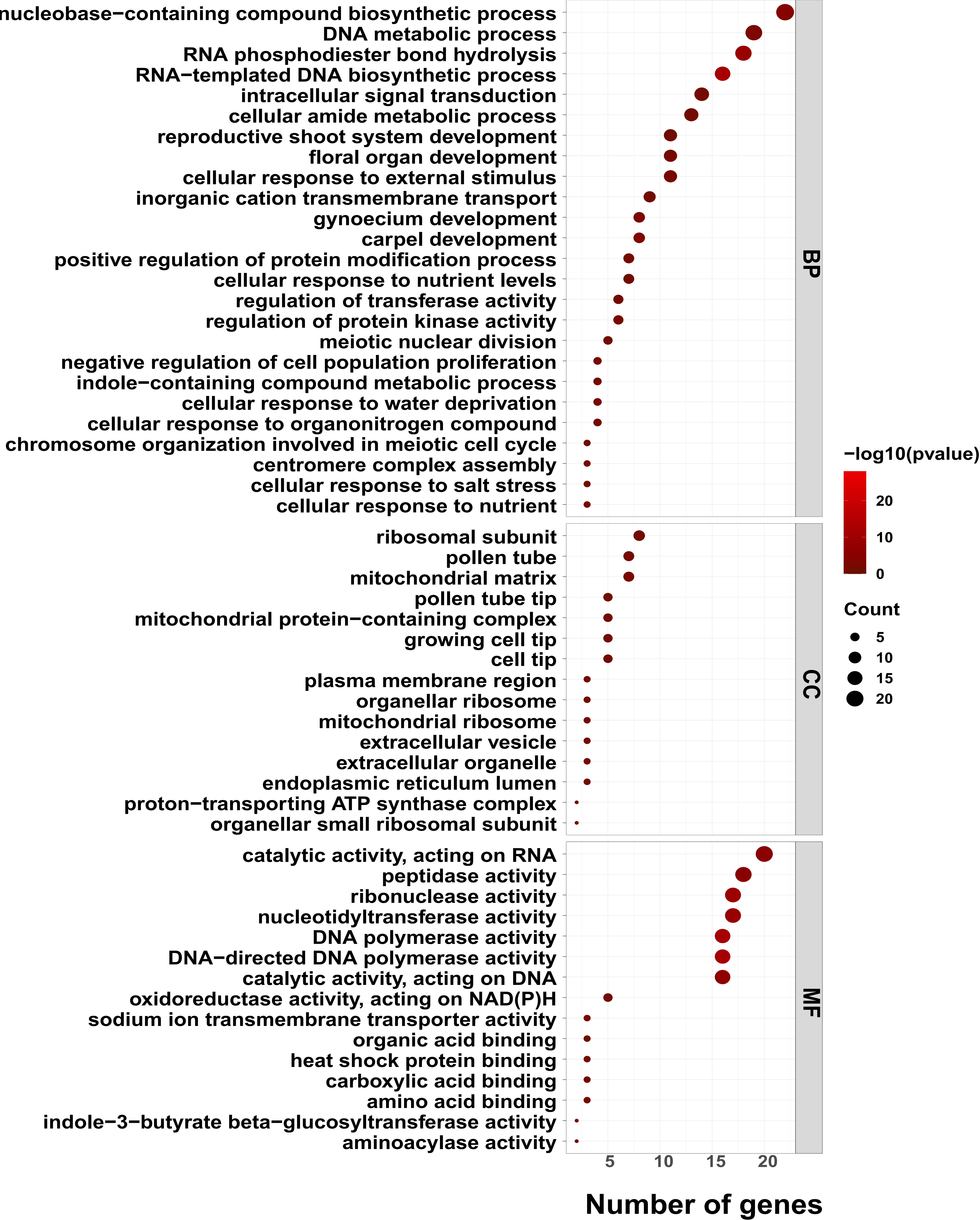

Supplement: Web_Material_uhad189 [file web_material_uhad189.zip › Figure S3.pdf]

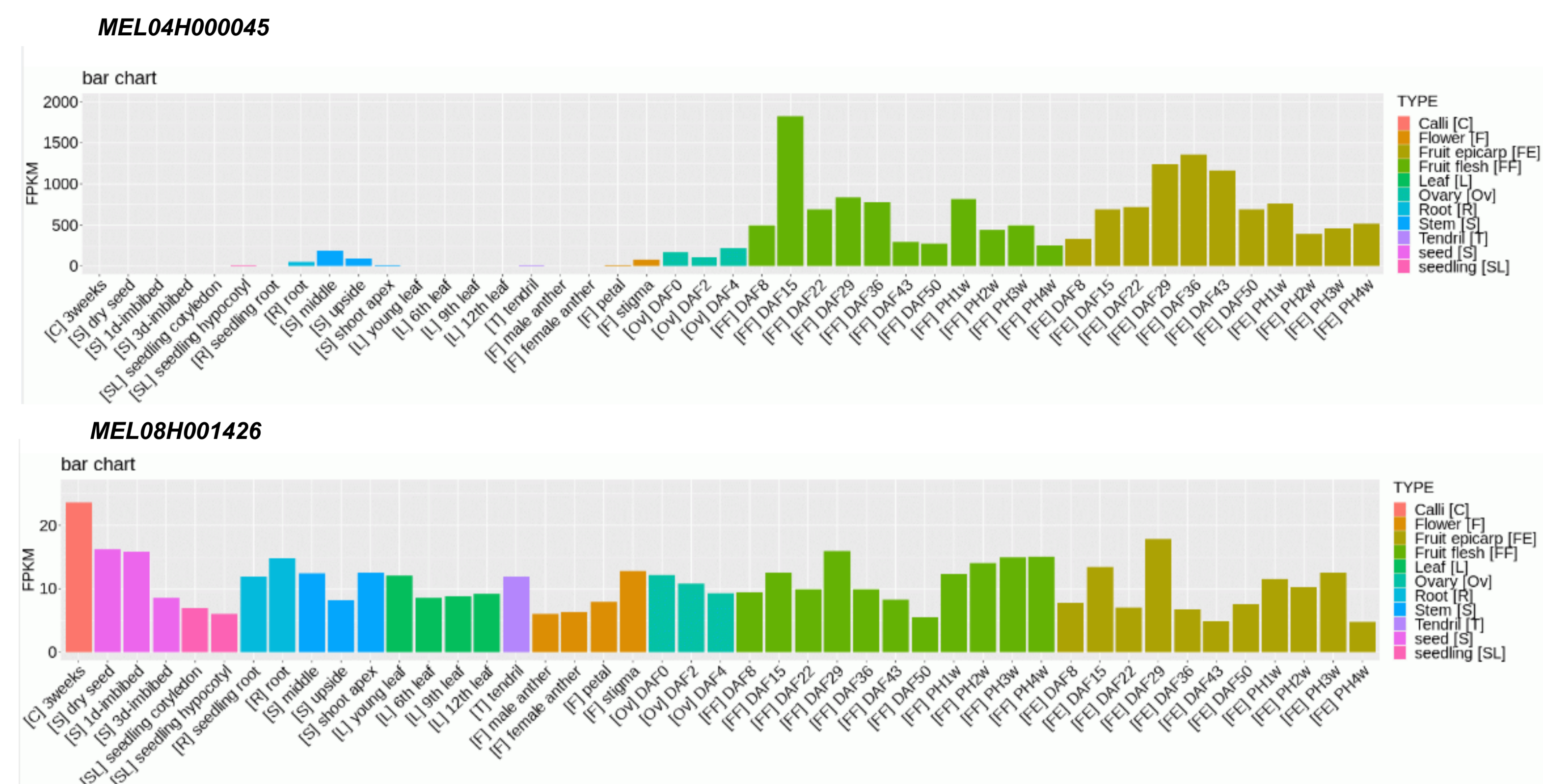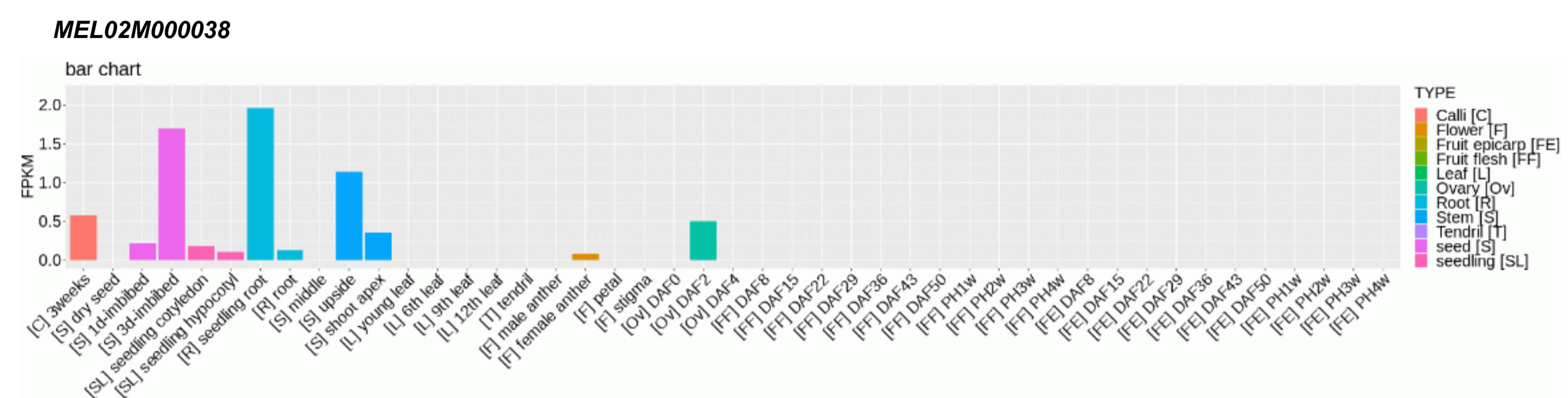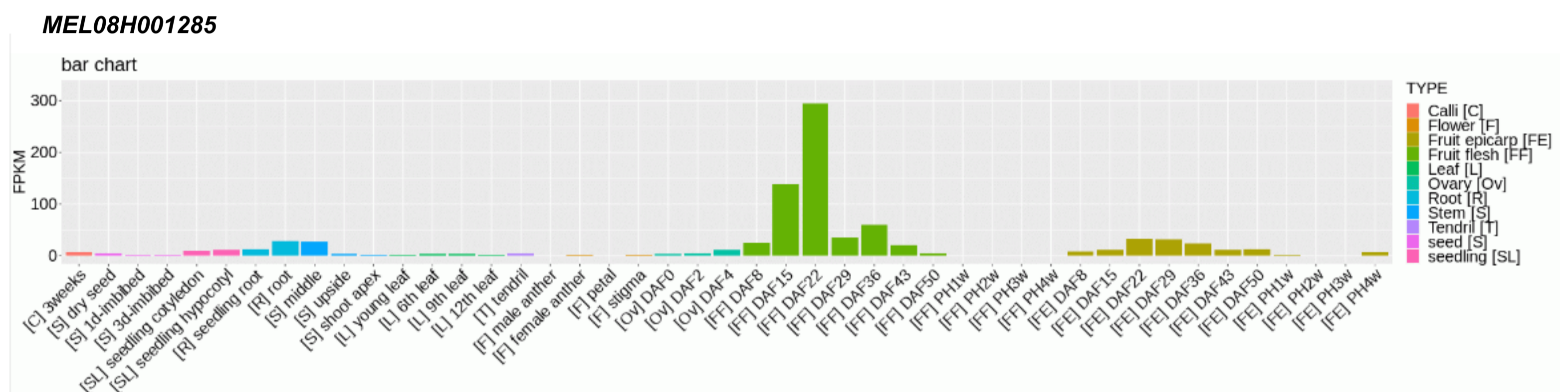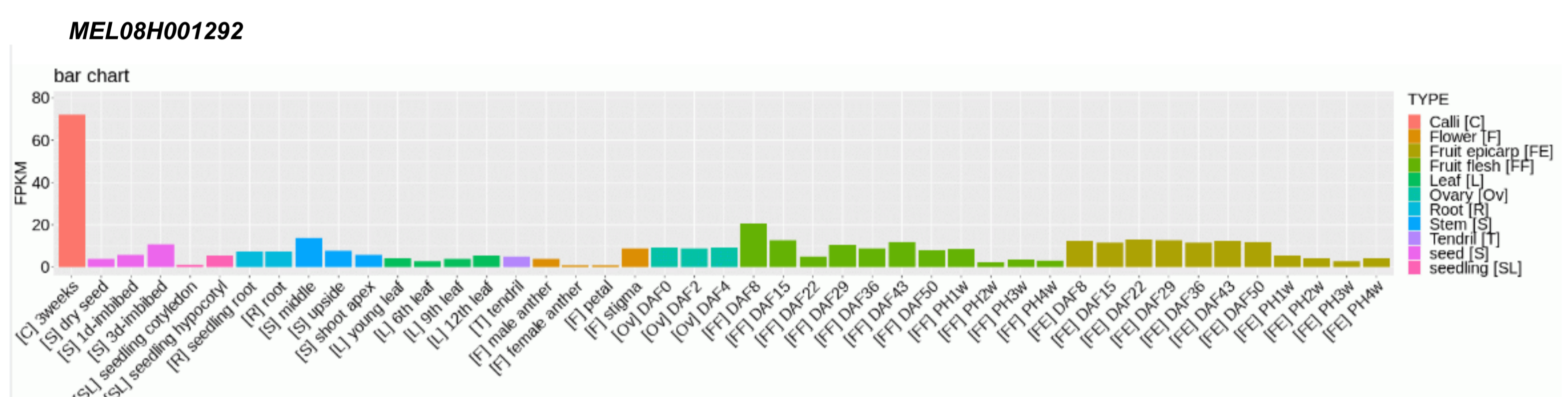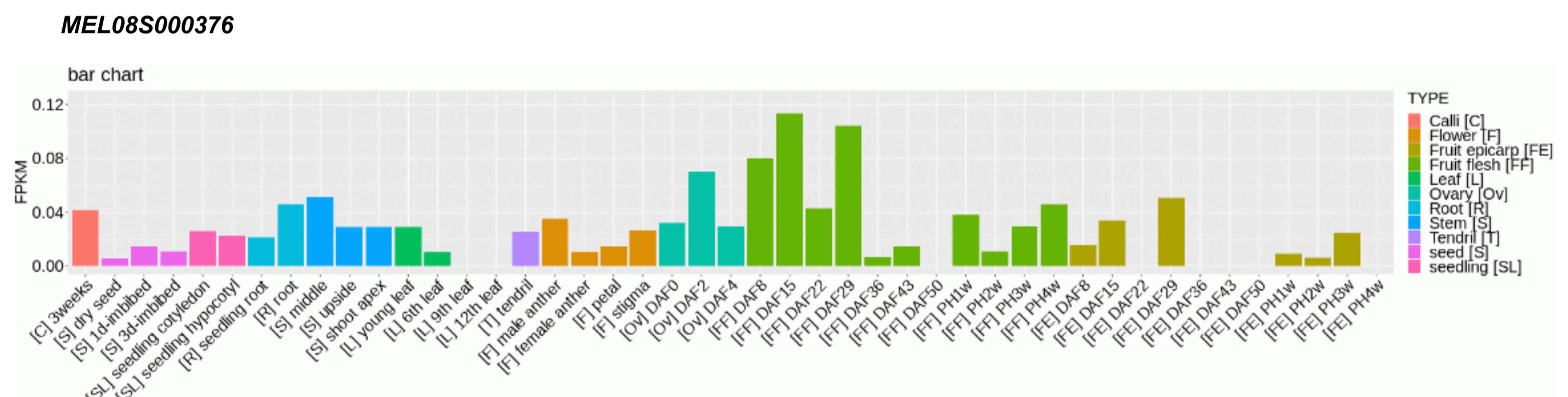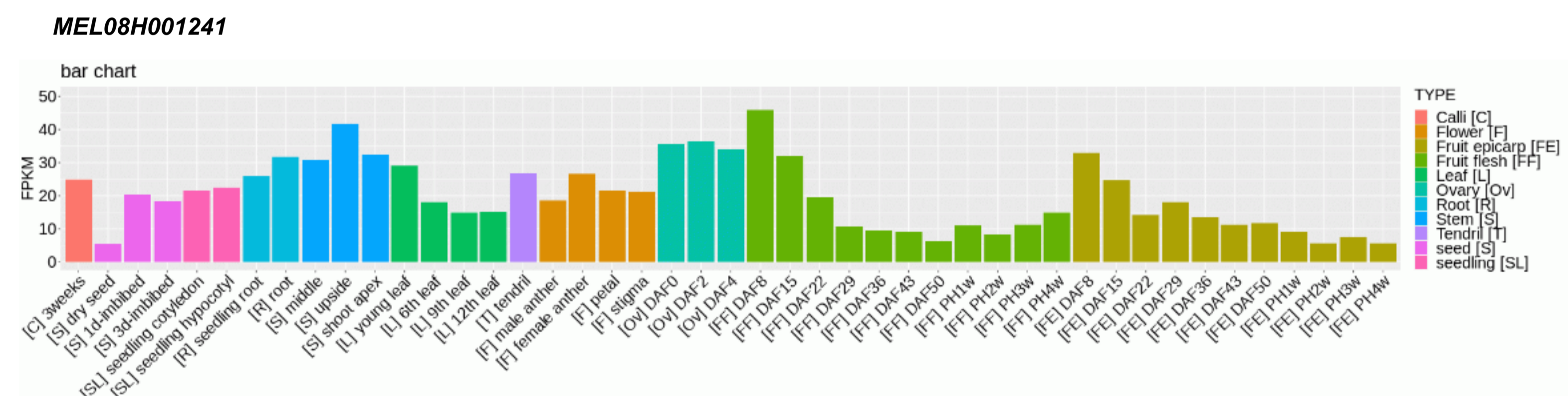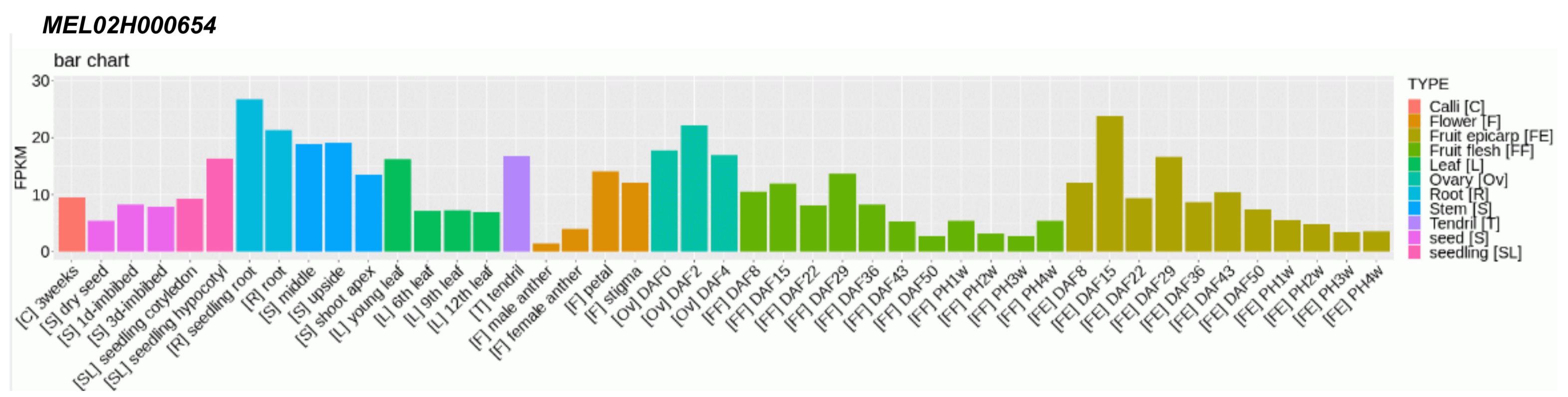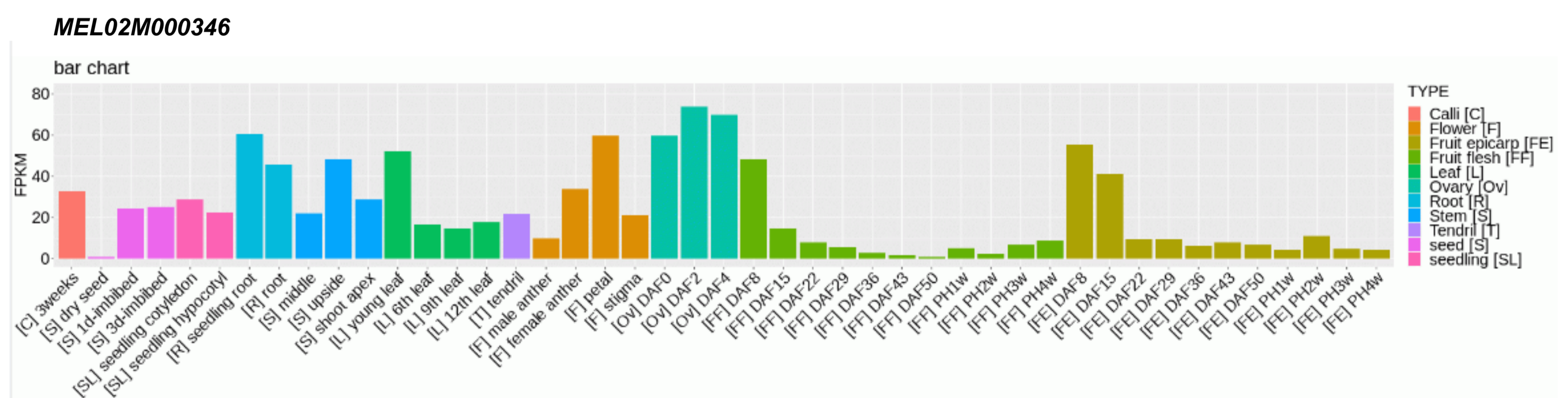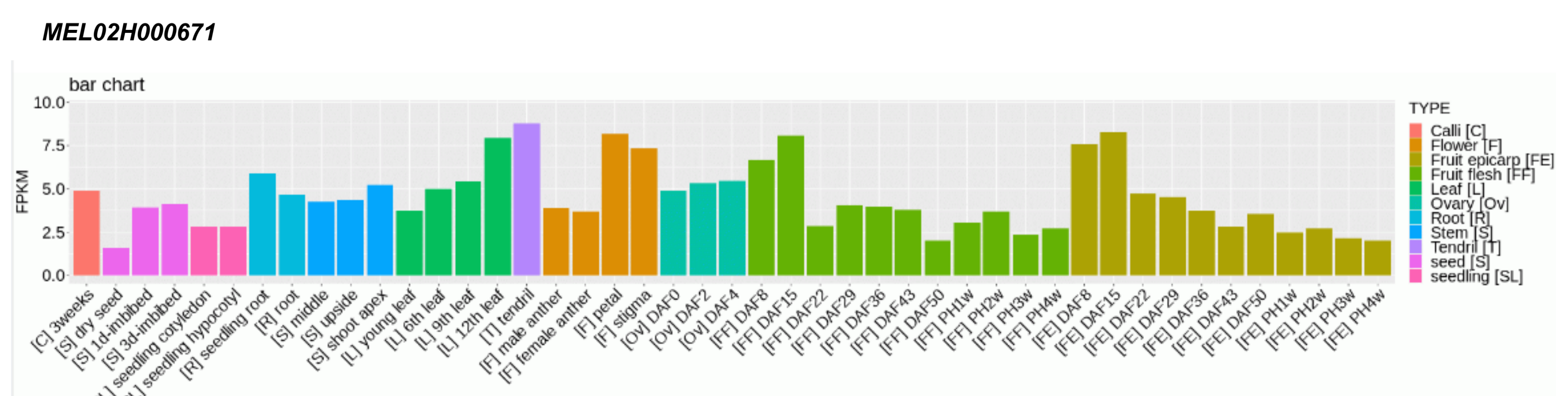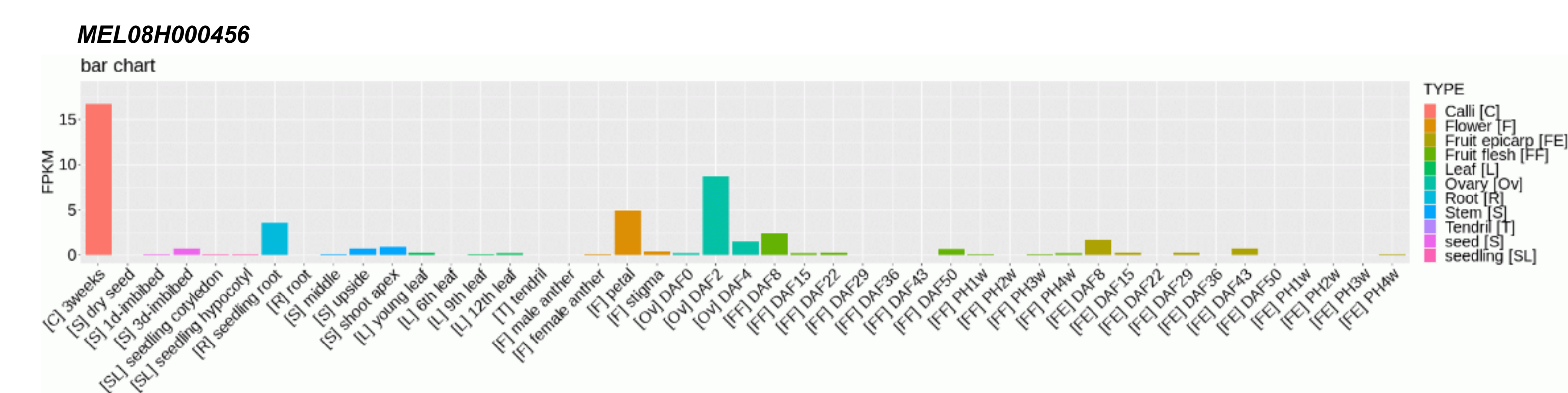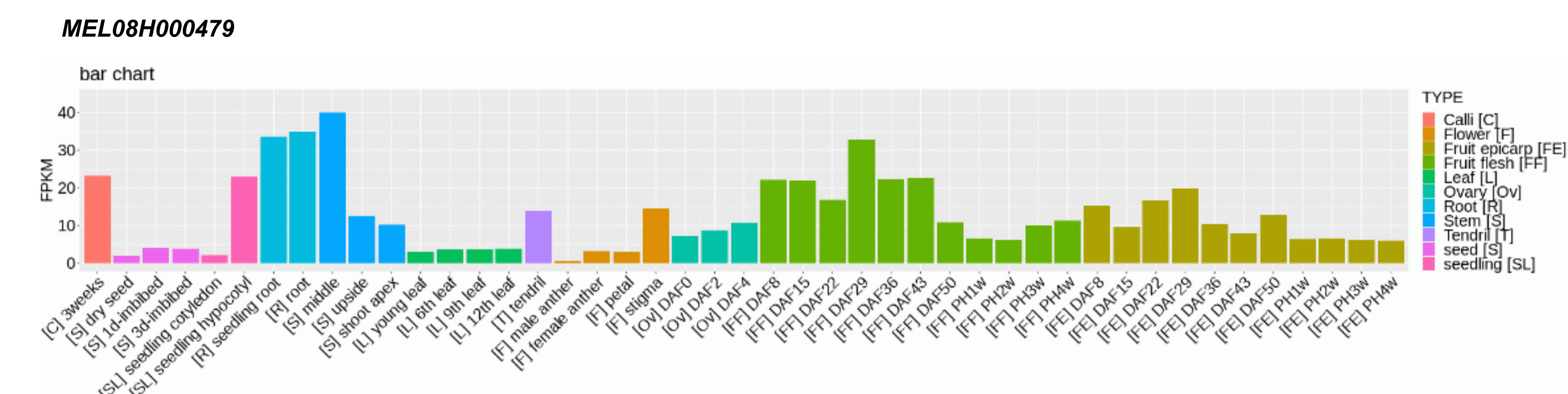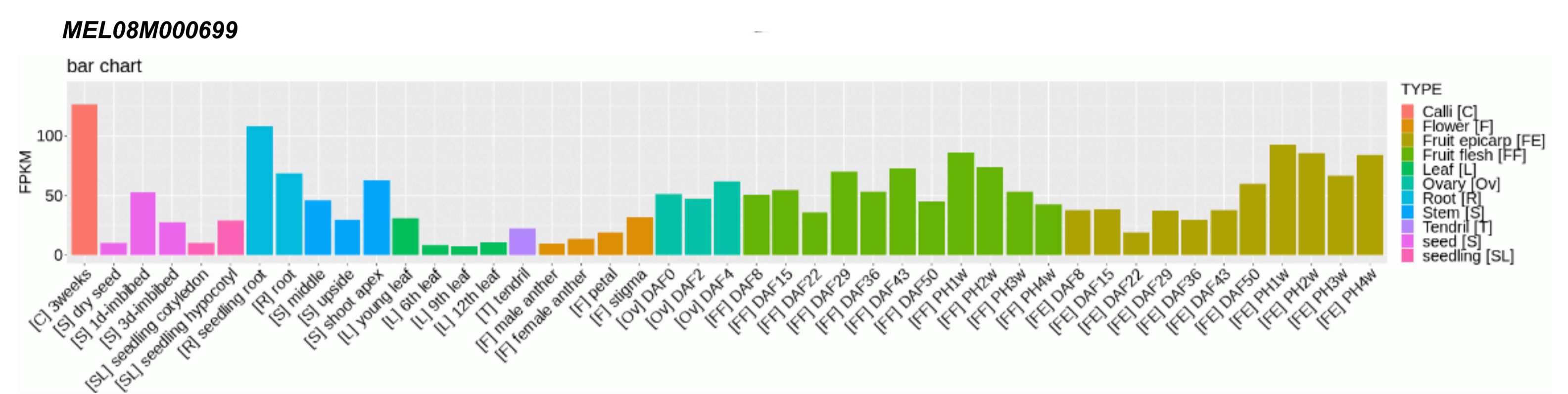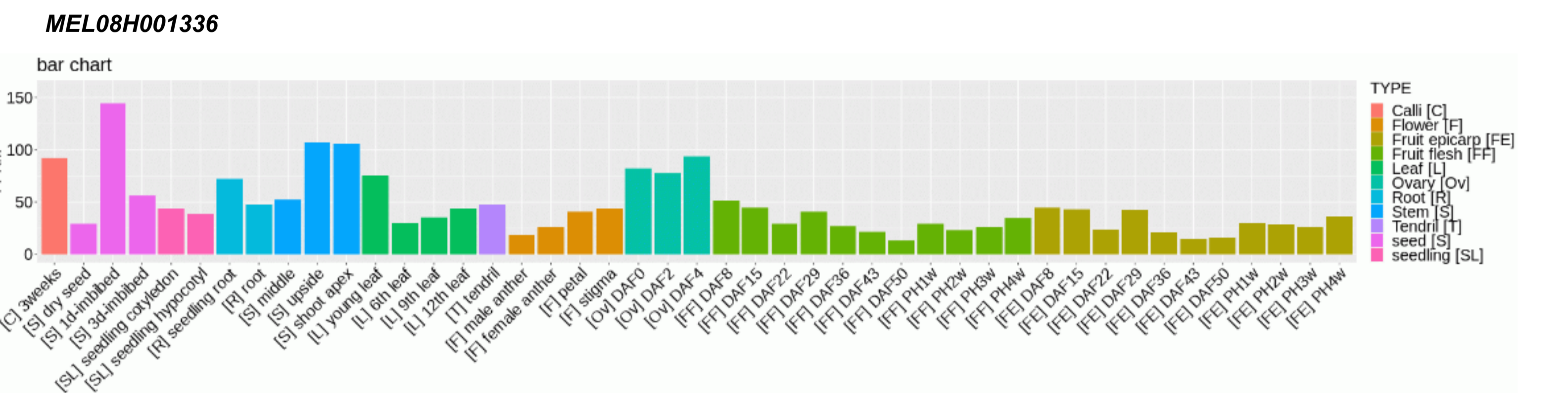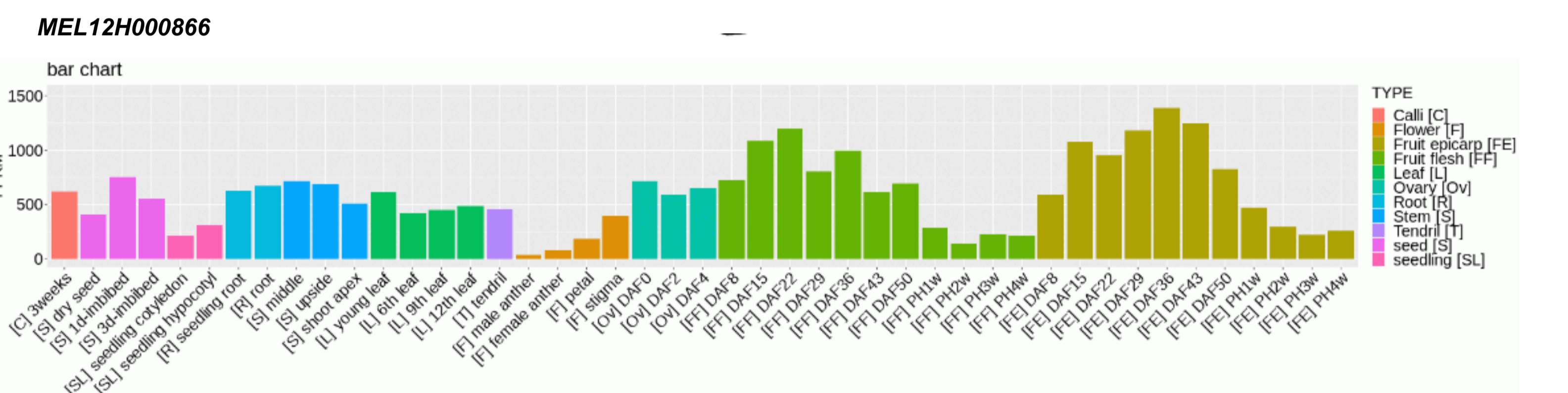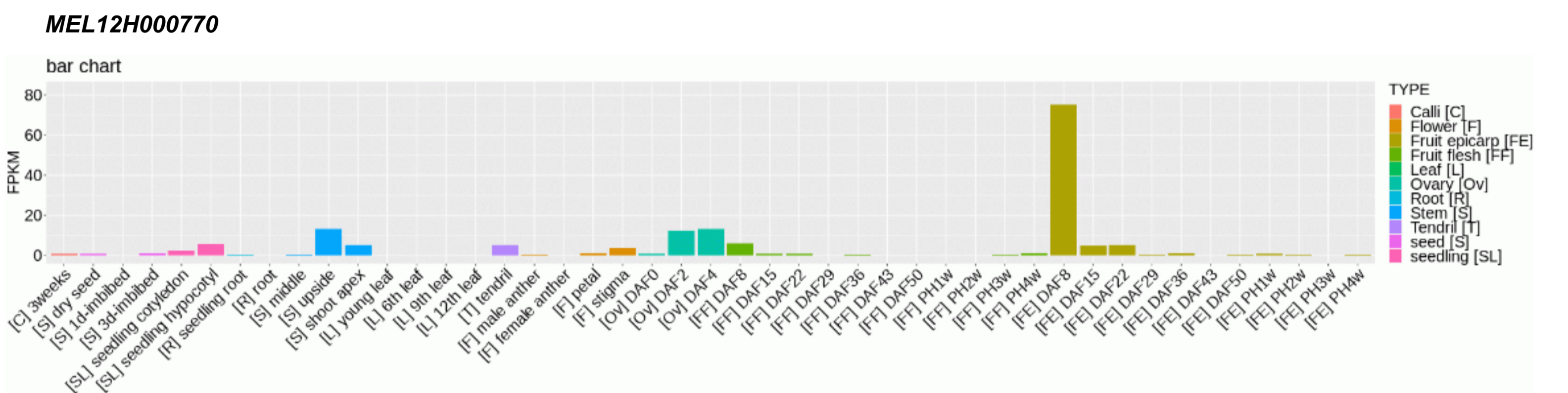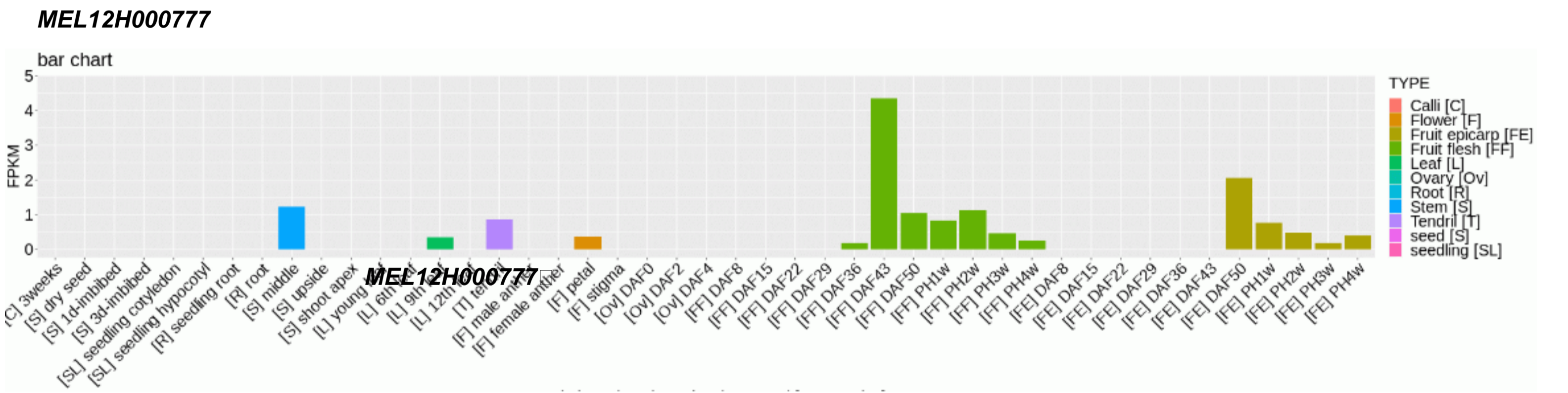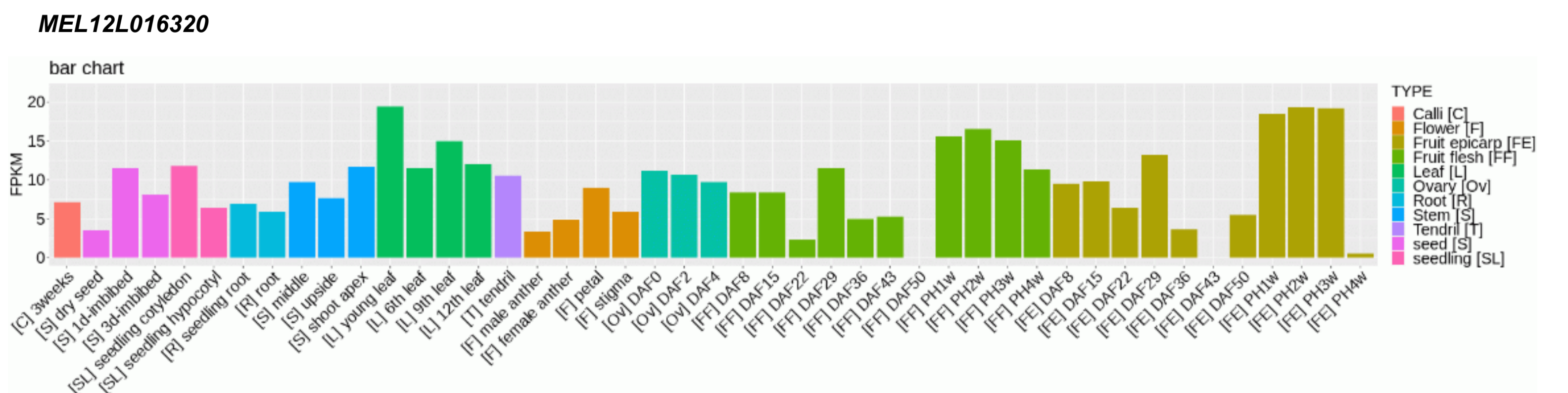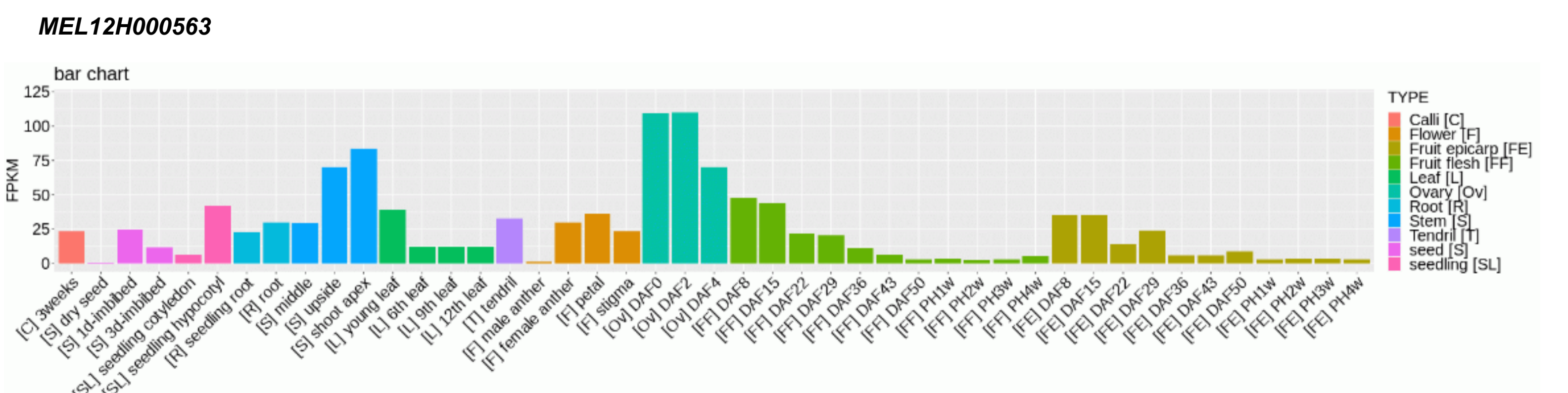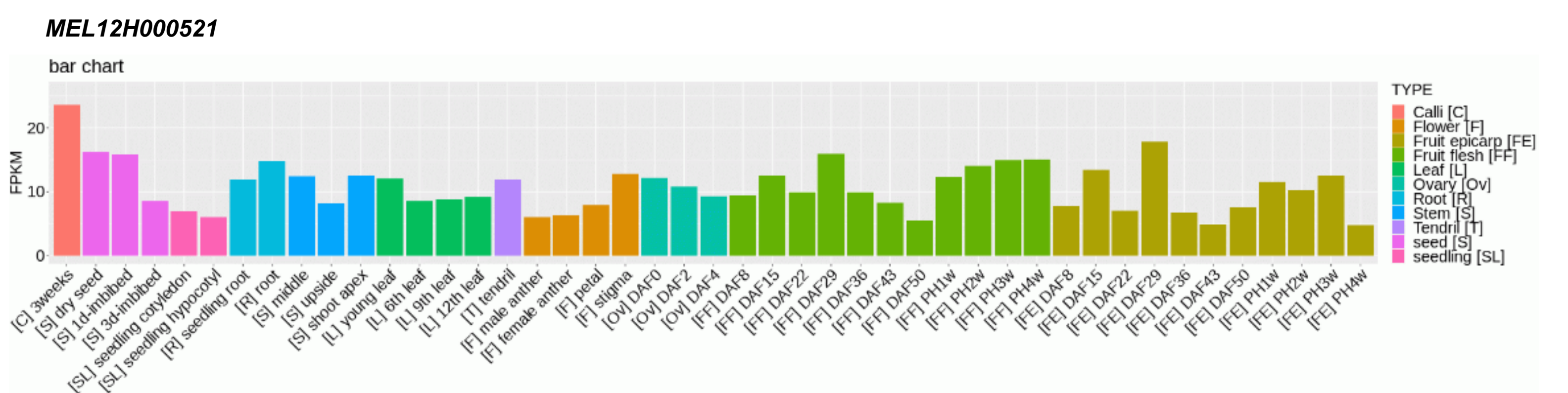

Supplement: Web_Material_uhad189 [file web_material_uhad189.zip › Figure S6.pdf]
