# Supplementary material for: Telomere-to-telomere genome assembly of melon (Cucumis melo L. var. inodorus) provides a high-quality reference for meta-QTL analysis of important traits
Source: Hortic Res. 2023 Sep 28;10(10):uhad189. doi: 10.1093/hr/uhad189 (PMC10615816; doi:10.1093/hr/uhad189)
Supplement: Web_Material_uhad189 [file Figure S5.pdf › Figure S5.pdf]

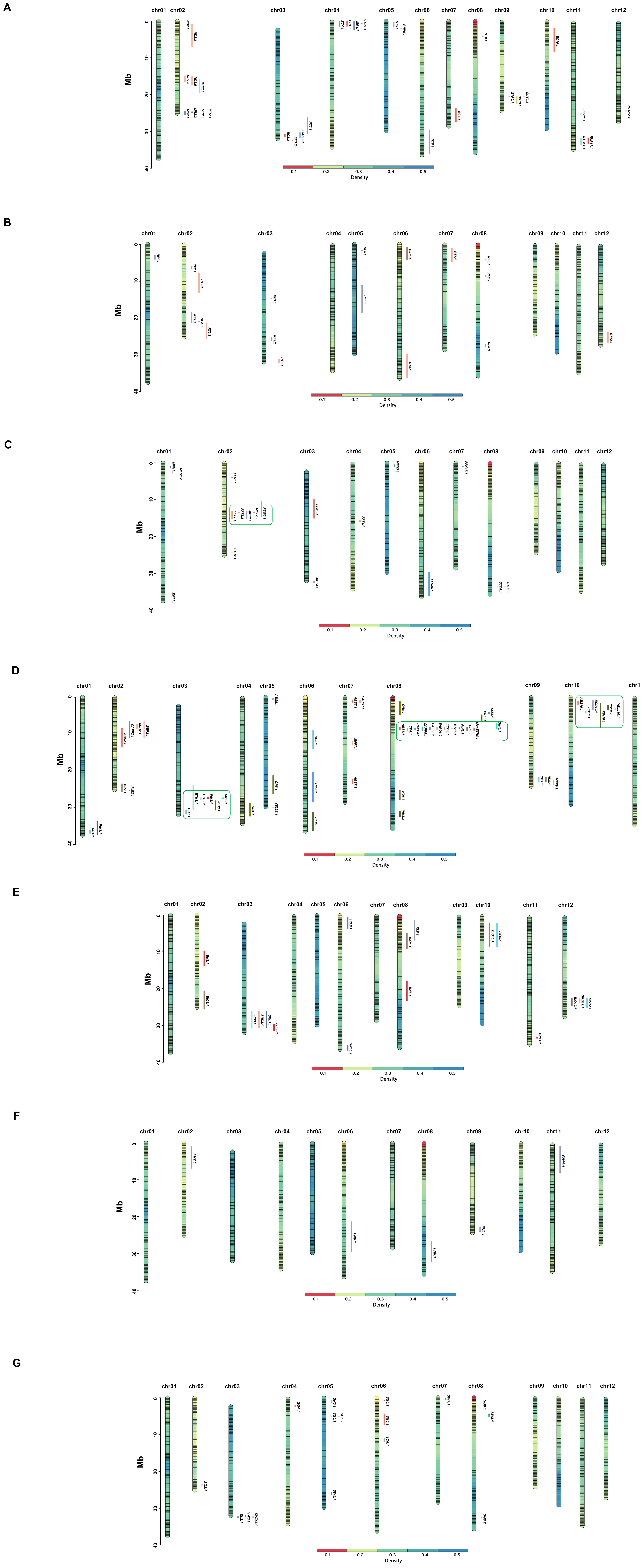

A
B
C
D
E
F
G
chr01
chr02
chr03
chr04
chr05
chr06
chr07
chr08
chr09
chr10
chr11
chr12
Mb
0
10
20
30
40
0.1
0.2
0.3
0.4
0.5
Density
